# Heuristic Optimization Algorithm of Black-Winged Kite Fused with Osprey and Its Engineering Application

**DOI:** 10.3390/biomimetics9100595

**Published:** 2024-10-01

**Authors:** Zheng Zhang, Xiangkun Wang, Yinggao Yue

**Affiliations:** 1 School of Information Engineering, Wenzhou Business College, Wenzhou 325035, China; 2 School of Intelligent Manufacturing and Electronic Engineering, Wenzhou University of Technology, Wenzhou 325035, China

**Keywords:** black-winged kite algorithm, osprey optimization algorithm, Cauchy mutation, metaheuristic algorithm, engineering application problems

## Abstract

Swarm intelligence optimization methods have steadily gained popularity as a solution to multi-objective optimization issues in recent years. Their study has garnered a lot of attention since multi-objective optimization problems have a hard high-dimensional goal space. The black-winged kite optimization algorithm still suffers from the imbalance between global search and local development capabilities, and it is prone to local optimization even though it combines Cauchy mutation to enhance the algorithm’s optimization ability. The heuristic optimization algorithm of the black-winged kite fused with osprey (OCBKA), which initializes the population by logistic chaotic mapping and fuses the osprey optimization algorithm to improve the search performance of the algorithm, is proposed as a means of enhancing the search ability of the black-winged kite algorithm (BKA). By using numerical comparisons between the CEC2005 and CEC2021 benchmark functions, along with other swarm intelligence optimization methods and the solutions to three engineering optimization problems, the upgraded strategy’s efficacy is confirmed. Based on numerical experiment findings, the revised OCBKA is very competitive because it can handle complicated engineering optimization problems with a high convergence accuracy and quick convergence time when compared to other comparable algorithms.

## 1. Introduction

The primary function of the swarm intelligence algorithm is to mimic the natural group behavior of fish, birds, animals, insects, and other populations. It does this by modeling the information sharing and exchange that occurs between members of the population and between members of the group behavior process [1,2,3]. Physical events or biological collective activity seen in nature serve as the basis for swarm intelligence optimization algorithms, which may be utilized to address function optimization issues [4,5]. It is practical, simple to use, and effective at resolving issues. It also offers fresh perspectives on how to resolve specific real-world application issues [6,7]. Complicated engineering issues with multipole values, complicated restrictions, high dimensionality, and nonlinearity are addressed using metaheuristic algorithms. Swarm-based algorithms, evolutionary algorithms, and physical phenomenon-based algorithms are the three categories of metaheuristic algorithms that draw inspiration from natural systems [8,9]. Over the past ten years, an infinite number of optimization algorithms have been developed as a result of the quick growth of swarm intelligence optimization algorithms and extensive study by multiple academics [10,11]. Examples of optimization algorithms based on predation behavior include the classical particle swarm optimization (PSO), the whale optimization algorithm (WOA), which explores and develops the population behavior of whales, and the grey wolf optimization (GWO), which bases its algorithm on how wolves surround, hunt, and attack prey during predation [12,13,14]. In contrast to the aforementioned algorithms, the black-winged kite algorithm (BKA) closely mimics the flight and predation behavior of black-winged kites in the wild thanks to its distinctive biological heuristic features [15]. The kite can easily adjust to changing conditions and target locations. The algorithm’s strong dynamic search capacity comes from mimicking this biological mechanism, which helps it adapt to the ever-changing optimization environment. Because of this, the technique has many uses in data mining, machine learning, engineering optimization, etc. [16].

The black-winged kite’s location is initialized in an overly random manner during the BKA optimization procedure. When updating an individual’s position through aggressive behavior, it heavily relies on the location of the previous generation black-winged kite, neglecting to account for exploration and development. Firstly, logistic chaotic mapping is used to initialize the population’s diversity, which improves the performance of the BKA. Second, the global exploration strategy of the osprey optimization algorithm in the first stage is used to substitute the position update formula of the individual attack behavior, improving the global search ability of the black-winged kite individual by combining individual abilities and natural factors. This is achieved by merging the osprey optimization algorithm (OOA) with the original black-winged kite algorithm. CEC2005, CEC2021, and three engineering examples are used to compare the proposed algorithm and BKA with eight existing intelligent algorithms. Next, the improved algorithm’s efficiency is verified.

The main contributions of this paper are as follows:

1. A detailed description of the optimization algorithm for black-winged kites is comprehensively provided.
2. A heuristic optimization algorithm (OCBKA) incorporating the osprey black-winged kite algorithm is proposed. The algorithm initializes the population through logistic chaotic mapping and combines the osprey optimization algorithm with the black-winged kite attack behavior, partially replacing the position update formula to improve the search performance of the algorithm.
3. It offers a creative solution to enhance black-winged kites’ ability to attack. Targeted replacement of partial position update methods is required in order to address the issue of excessive reliance on the previous generation of black-winged kites for a partial position update during their attack behavior.
4. A comprehensive experimental comparison and analysis are conducted on the improved algorithm.

## 2. Black-Winged Kite Algorithm

Wang Jun et al. presented the black-winged kite optimization algorithm (BKA), a revolutionary metaheuristic algorithm, in March 2024. The black-winged kite draws inspiration from its survival strategy. They have amazing hovering skills and can hunt with surprising success. They eat insects, birds, reptiles, and small mammals. Based on the black-winged kite’s travel patterns and hunting skills, an algorithmic model was created [17].

### 2.1. Aggressive Behavior

After silently watching its target for a time and angling its wings and tail to match the wind speed, the black-winged kite, a small creature on the grassland, would quickly drop and attack. The black-winged kite was simulated in two assault states during the global exploration phase of the BKA. In the first, while the kite hovers in midair, it continually adjusts its position to race to the target at the ideal assault angle. In the second, the kite hovers in midair and continually looks for targets, hitting them when it finds the one that is easiest to take down. The mathematical model of the attack behavior is expressed as:

(1) $$x_{i,j}^{t+1}=\left\{\begin{array}{c} x_{i,j}^{t}+n(1+\sin(r))\times x_{i,j}^{t}\;p<r\\ x_{i,j}^{t}+n\times (2r-1)\times x_{i,j}^{t}\;else \end{array}\right.$$

(2) $$n=0.05\times e^{-2\times {\left(\frac{t}{T}\right)}^{2}}$$

Among them, $x_{i,j}^{t}$ and $x_{i,j}^{t+1}$, respectively, represent the position of the *i*-th black-winged kite in the *j*-th dimension in the *t*-th and (*t* + 1)-th iterations, *r* is a random number ranging from 0 to 1, *p* is a constant value of 0.9, *T* is the total number of iterations, and *t* is the current number of iterations.

### 2.2. Migration Behavior

The BKA mimics the complicated behavior of black-winged kite migration during the exploitation phase. Bird migration in nature is governed by several variables, including climate and food availability. Lead birds are frequently involved in migrations, and their ability to navigate is essential to the migration’s success. In the BKA, the Leader strategy is combined with the migratory characteristics of birds: if the current population’s fitness value is lower than that of the random population, it suggests that it is unfit to lead the current population forward, and it will lose its leadership and join the migratory population; on the other hand, if the current population’s fitness value is higher than that of the random population, it will drive the direction of population migration. The plan makes it possible to choose outstanding leaders in a dynamic way, which guarantees the migration’s success.

The mathematical model expression for the migration behavior of black-winged kites is as follows:

(3) $$x_{i,j}^{t+1}=\left\{\begin{array}{c} x_{i,j}^{t}+C(0,1)\times (x_{i,j}^{t}-L_{j}^{t})\;\;\;\;F_{i}<F_{ri}\\ x_{i,j}^{t}+C(0,1)\times \left(L_{t}^{j}-m\times x_{i,j}^{t}\right)\;\;\;\;else \end{array}\right.$$

(4) m=2×sin(r+π/2)

Among them, $L_{j}^{t}$ represents the score of the leader of the black kite in the *j*-th dimension in the *t*-th iteration so far, $x_{i,j}^{t}$ and $x_{i,j}^{t+1}$ represent the position of the *i*-th black-winged kite in the *j*-th dimension in the *t*-th and (*t* + 1) iterations, *F_i_* represents the fitness value of any individual in the *t*-th iteration, *F_ri_* represents the fitness value of any black-winged kite in the *t*-th iteration, and *C*(0,1) represents the Cauchy mutation, defined as follows:

(5) $$f(x,\delta ,\mu )=\frac{1}{\pi }\frac{\delta }{\delta^{2}+{\left(x-\mu \right)}^{2}},\;\;\;\;-\infty <x<\infty$$

When δ = 1 and μ = 0, the expression for the Cauchy mutation is:

(6) $$f(x,\delta ,\mu )=\frac{1}{\pi }\frac{1}{x^{2}+1},\;\;\;\;-\infty <x<\infty$$

## 3. Improved Strategy of Black Kite Heuristic Algorithm with Fusion of Osprey

Heuristic algorithm research has traditionally focused on how to increase the global search range in order to prevent slipping into local optima. This is because maintaining a healthy balance between global and local search is crucial to algorithm optimization. The BKA still suffers from the imbalance between global search and local development skills, and it is prone to local optimization even if it combines the advantages of Cauchy mutation and the Leader approach to enhance the algorithm’s optimization capacity. Thus, this study proposed the OCBKA, initialized the population through logistic chaotic mapping, and fused the global exploration strategy of the osprey optimization algorithm in the first stage to improve the search performance of the algorithm. This was carried out in order to balance the global search and local development capabilities of the BKA.

### 3.1. Population Initialization Based on Logistic Mapping

In essence, the swarm intelligence optimization algorithm is a random search optimization method, and it is commonly represented by the PSO algorithm. Setting the algorithm’s required parameters should come first in application, followed by the population’s initialization process [18]. The beginning population is often generated by the random initialization operation; however, because of the unequal distribution of people in the starting population, the convergence performance of the algorithm is fundamentally constrained. After comparison and analysis, we decided to use logistic mapping for population initialization, based on the properties of chaotic mapping (randomness, ergodicity, and regularity) that satisfy the initial population criteria [19]. Equation (7) displays the equation of the chaotic sequence generated by logistic mapping. Among them, $Y_{i+1}$ is the chaotic sequence, and μ is the branch parameter, which takes values in $\left(0,4\right]$. The larger μ, the higher the chaos. When 3.5699456 < μ ≤ 4, the logistic map has chaotic properties:

(7) $$Y_{i+1}=\mu Y_{i}(1-Y_{i}),Y_{i}\in (0,1)$$

**Figure 1:**
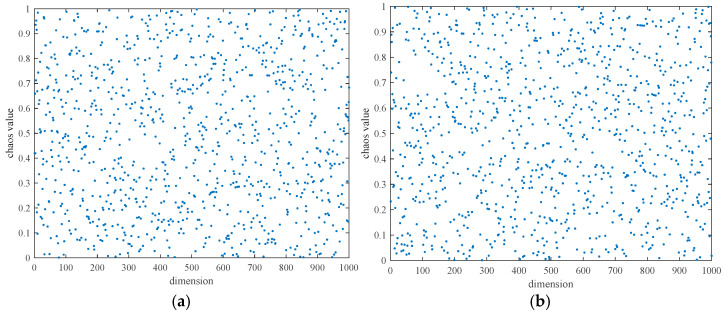
Distribution diagram of numerical values. (**a**) Logistic mapping generation. (**b**) Generation of random numbers.

The distribution diagram for 1000 series values generated using logistic mapping and widely used random numbers, respectively, is displayed in Figure 1. The distribution of chaotic sequence values between 0 and 1 produced by logistic mapping was more uniform than that of sequence values produced by regular random numbers, as can be seen in Figure 1. Its incorporation into the population’s initialization process can offer the algorithm a high-quality search space, which helps to increase the method’s accuracy of convergence.

This article introduced the population initialization operation of logistic mapping for position initialization, as shown in Equation (8), where *x_up_* and *x_lb_* are the upper and lower bounds of individual positions, *Y_i_* is the chaotic sequence value generated by the logistic mapping on the corresponding individual, and $x_{i,j}$ is the position of the *i*-th black-winged kite in the *j-*th dimension:

(8) $$x_{i,j}=x_{lb}+(x_{up}-x_{lb})\times Y_{i}$$

### 3.2. Improved Black-Winged Kite Algorithm with Fusion of Osprey

The original algorithm’s attack behavior is overly dependent on the location of the black-winged kite from the previous generation, and the search degree of the optimization space is insufficient, which makes it easy for the algorithm to settle into a local optimum and makes it difficult to strike a good balance between local development and global exploration [20,21]. Consequently, the attack behavior position update formula of the original black-winged kite method was replaced with the global exploration strategy of the osprey optimization algorithm in the first stage [22,23]. The black-winged kite’s degree of exploration in uncharted territory was increased when one of the meals was randomly located and attacked, enhancing the algorithm’s local development and global optimization to strike a healthy equilibrium. The following is the updated attack behavior strategy: 

(1) The position of the “underwater fish swarm” may be described as follows [24] in order to find additional black-winged kites in the search space that have better positions (lower objective function values):

(9) $$FP_{i}=\left\{X_{k}|k\in \left\{1,2,\cdots ,N\right\}\wedge F_{k}<F_{i}\right\}\cup \left\{X_{best}\right\}$$

In the formula, *X_k_* is the position vector of the *k*-th black-winged kite, *X_best_* is the optimal individual position vector for black-winged kites, while *F_k_* and *F_i_* are the fitness values of the *k*-th and *i*-th black-winged kites, respectively. The parameter *N* is the population size of black-winged kites.

(2) The new attack behavior formulas after integrating the osprey optimization algorithm are shown in Equations (10) and (11) [25]:

(10) $$x_{i,j}^{t+1}=\left\{\begin{array}{c} x_{i,j}^{t}+r\cdot (SF_{i,j}-I_{i,j}\cdot x_{i,j}^{t})\;\;\;\;\;\;p<r\\ x_{i,j}^{t}+n\times (2r-1)\times x_{i,j}^{t}\;\;else \end{array}\right.$$

(11) $$n=0.05\times e^{-2\times {\left(\frac{t}{T}\right)}^{2}}$$

Among them, $x_{i,j}^{t}$ and $x_{i,j}^{t+1}$, respectively, represent the position of the *i*-th black-winged kite in the *j*-th dimension in the *t*-th and (*t* + 1)-th iterations, *r_i,j_* is random numbers between 0 and 1, and *p* is a constant of 0.9. *SF_i_* is the position vector corresponding to an individual randomly selected from the fish group corresponding to the *i*-th fish eagle (i.e., *FP_i_*) and *SF_i,j_* is the value of *SF_i_* in the *j*-th dimension. The parameter *r_i,j_* is random numbers within the range of (0,1) and the parameter *I_i,j_* is the value from {0,1}.

Local exploration benefits from this procedure. In order to improve the optimization efficiency, the candidate solution updating process can thereby improve exploration capabilities.

### 3.3. The OCBKA Flowchart and Pseudocode

**Figure 2:**
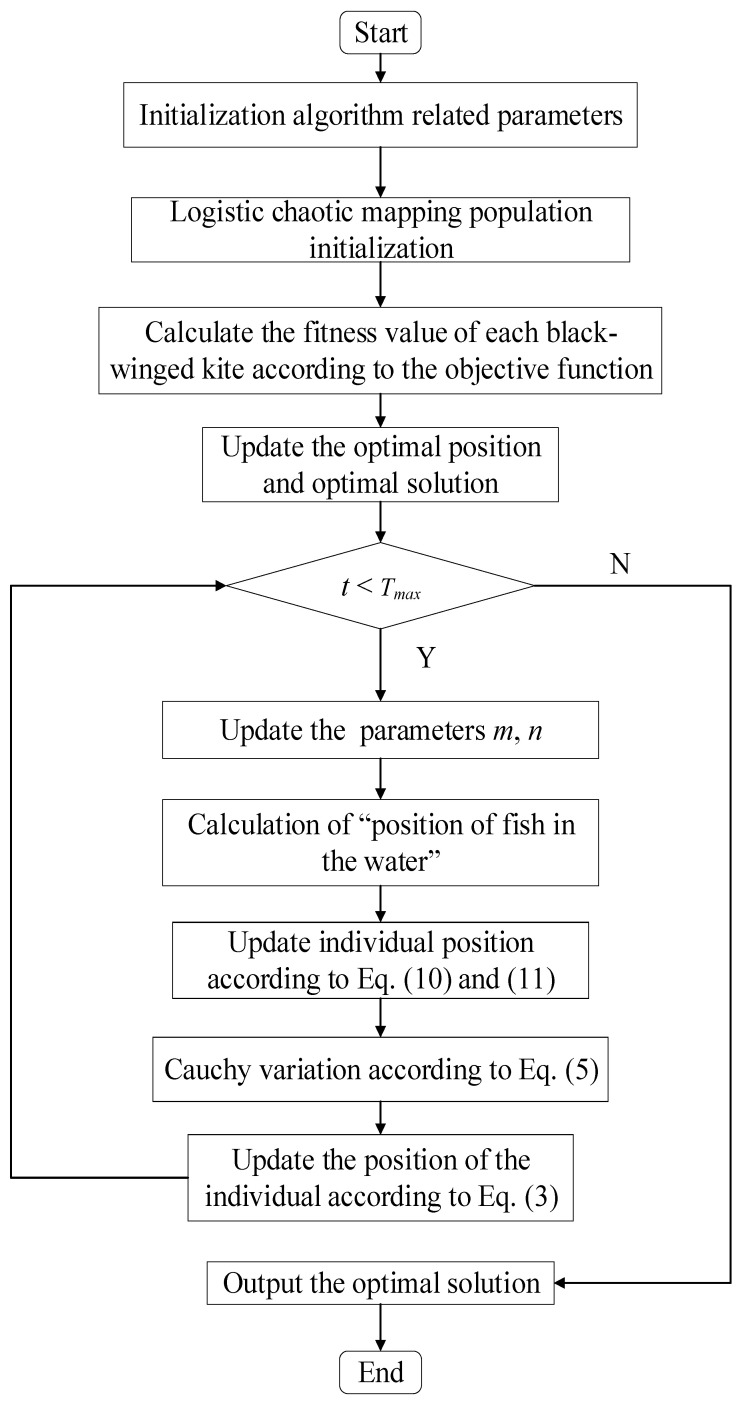
The workflow of the OCBKA.

The OCBKA’s pseudocode is shown in Algorithm 1, and Figure 2 depicts the algorithm’s process.
**Algorithm 1.** Pseudocode of OCBKA Inputs: the maximum number of iterations is *T*, the size of the population is *N*. Output: optimal position, *X_best_*, and fitness value, *F*(*X_best_*).1.Initialize the population according to Equation (8) and specify the relevant parameters;2.    **While** (*t* ≤ *T*) **do**3.    Calculate the fitness value, F(*X_i_*), for each individual in the population, and record the current optimal solution and optimal position;4.    Update the values of parameters m and n.;5.    **for** i = 1 to *N* **do**6.        Update the position of the “underwater fish school” according to Equation (9);7.        **if** *r* < *p* **do**8.            update the current individual position according to Equation (10);**9.**         **else if**10.            update the current individual position according to Equation (10);**11.**        **end if**12.        perform Cauchy mutation according to Equation (5);13.        **if** *F*(*X_i_*) < *F*(*X_ri_*) **do**14.            update the current individual position according to Equation (3);**15.**         **else if**16.            update the current individual position according to Equation (3);**17.**         **end if****18.**     **end for**19.    **if** the position of the newly generated black-winged kite population is better than that of the original population, **do**20.            update the position of the black-winged kite population;**21.**     **end if;**22.*t* = *t* + 1;**23.** **end While**24.return *X_best_* and *F*(*X_best_*).

### 3.4. Time Complexity Analysis

An essential metric for assessing an algorithm’s efficiency is its time computational complexity [26]. The maximum number of iterations (T), population size (N), and dimension (D) are strongly connected with the position initialization, fitness value computation, and position update components that make up the BKA’s time computational complexity. Consequently, the BKA’s temporal computational complexity is as follows: (1) it takes O(N*D) time to initialize the population, (2) it takes O(N) to compute the fitness value, and (3) it takes O(N*D*T) to update the location. As a result, the BKA’s overall time complexity is O*N*D*T, excluding low-order terms.

The fish eagle exploration method is used in lieu of some of the black-winged kite assault behavior in the OCBKA, which does not add to the time complexity of the BKA. As a result, the OCBKA’s time complexity is O*N*D*T, the same as the BKA.

## 4. Experimental Comparison and Result Analysis

To verify the convergence and optimization capabilities of the algorithm, as well as to showcase its effectiveness and progress, the CEC2005 test suite was used to assess the OCBKA function’s performance. The exact details of the test suite are displayed in Table 1. It has twenty-three functions total, grouped into four categories: basic multimodal functions (F8~F13), compound functions (F14~F23), and unimodal functions (F1~F7). First, OCBKA and BKA were contrasted, and the efficacy of the technique that OCBKA proposed was confirmed. Second, to confirm the progress made by OCBKA, it was compared to eight competitive heuristic algorithms that have been proposed recently. These include particle swarm optimization (PSO) [27], grey wolf optimizer (GWO) [28], Harris hawk optimization (HHO) [29], artificial gorilla troops optimizer (GTO) [30], snake optimizer (SO) [31], dung beetle optimizer (DBO) [32], golden jackal optimizer (GJO) [33], and subtraction-average-based optimizer (SABO) [34]. The analysis of the convergence accuracy and speed of the various algorithms was performed to confirm the progress of the OCBKA.

### 4.1. Experimental Setup

The Intel (R) Core (TM) i5-11400H @ 2.70 GHz processor, MATLAB R2022b, and Windows 11 operating system provided the experimental environment for the completion of the numerical experiment. In the logistic mapping in the OCBKA, we set the miu parameter to 3. The comparison’s other algorithmic control settings were in line with the appropriate original references.

Each method had a population size of N = 30, a dimension of D = 30, and an evaluation frequency of 30,000 times in order to maintain fairness. To demonstrate the correctness and stability of the algorithm, it was independently run thirty times. The ideal value, standard deviation, average value, and amount of time needed for each test function solution were noted. The standard deviation of the optimum value derived from the past 10 iterations was computed during the thirty-first run of each algorithm. The procedure really converged in 1000 iterations if the standard deviation was less than 0.01 because that suggests that the ideal value had not changed substantially. If so, it was shown as Yes; if not, it was indicated as No.

### 4.2. Comparative Experiment with Classical Swarm Intelligence Algorithm

**Figure 3:**
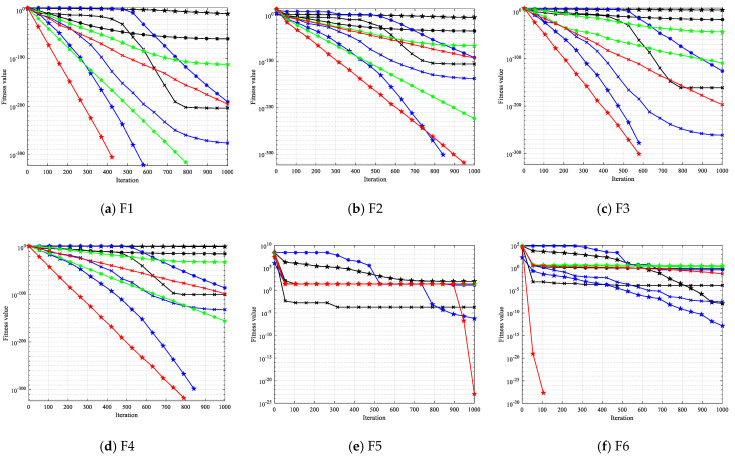

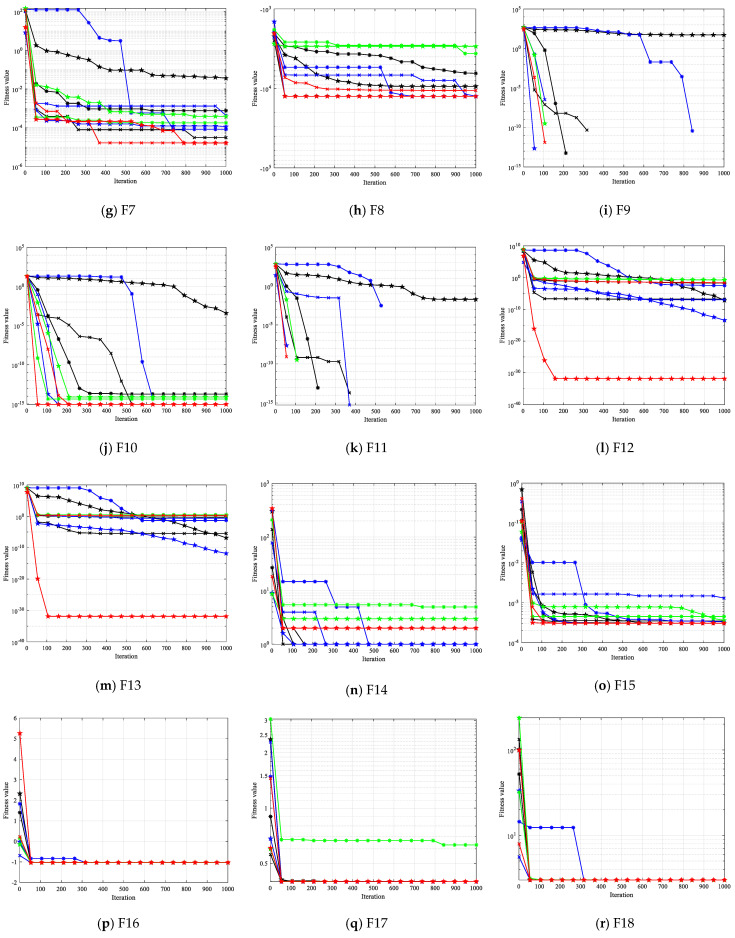

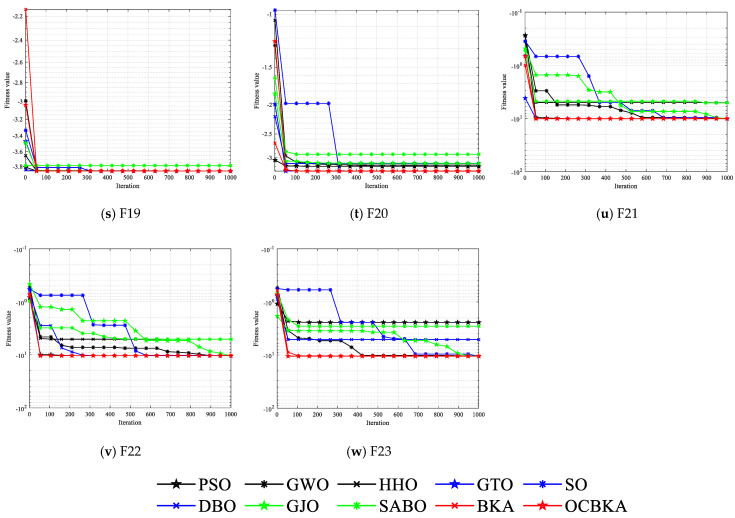
Iteration curves of the different improved algorithms.

In this section, the OCBKA was compared against eight intelligent optimization algorithms—the BKA, PSO, GWO, HHO, GTO, SO, DBO, GJO, and SABO—which have demonstrated significant competitiveness in the last several years. Table 2 displays the experimental findings, and the best outcomes are shown as bolded data. Figure 3 displays the iterations’ average convergence curves.

The data in Table 2 show that, in comparison to other optimization algorithms, the algorithm suggested in this paper found the best solution for solving all unimodal functions except F7, and that, in solving the F7 function, the mean value and standard deviation were the lowest, indicating that the improved algorithm had good stability. It was demonstrated that the modified black-winged kite method had a higher global search and development ability since the unimodal function only had one solution. When solving six multimodal functions, the OCBKA method yielded the best result with the maximum convergence accuracy. When it came to mixed function solving, OCBKA came out on top in F14, F15, F16, and F18 function solving, as well as achieving the top-three optimum solutions in other functions, but with a little lower accuracy than the best accuracy algorithm. This demonstrated the algorithm’s great optimization capabilities and how effectively the new motion mode of the algorithm can be controlled in terms of both convergence speed and accuracy. The fastest running times were achieved by PSO and SO, but their accuracy and speed convergence rates were relatively sluggish. Even though OCBKA took a bit longer to operate than BKA, it provided far faster and more accurate convergence. According to the no-free-lunch idea, we may tolerate the low time cost in exchange for a significant improvement in optimization performance. Upon examining the algorithm’s convergence, we discovered that the algorithm could effectively converge on the great majority of test functions when the maximum convergence frequency was set to 1000 times. Its convergence accuracy can, therefore, be compared and assessed appropriately.

Figure 3 illustrates that the convergence curves for the F9 and F11 functions only had one point for the OCBKA method. The enhanced OCBKA had a decent convergence speed, further demonstrating the usefulness of the revised approach. The reason for this is that the OCBKA’s convergence speed was too rapid.

### 4.3. Further Comparative Experiments of Algorithms

**Figure 4:**
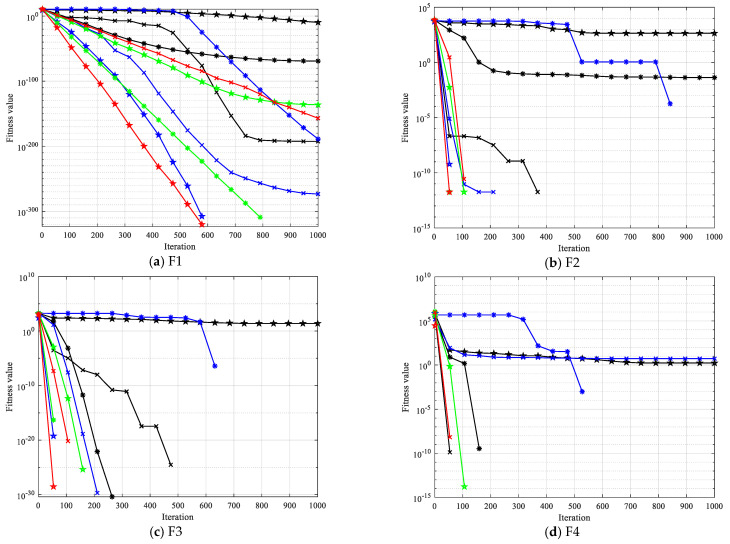

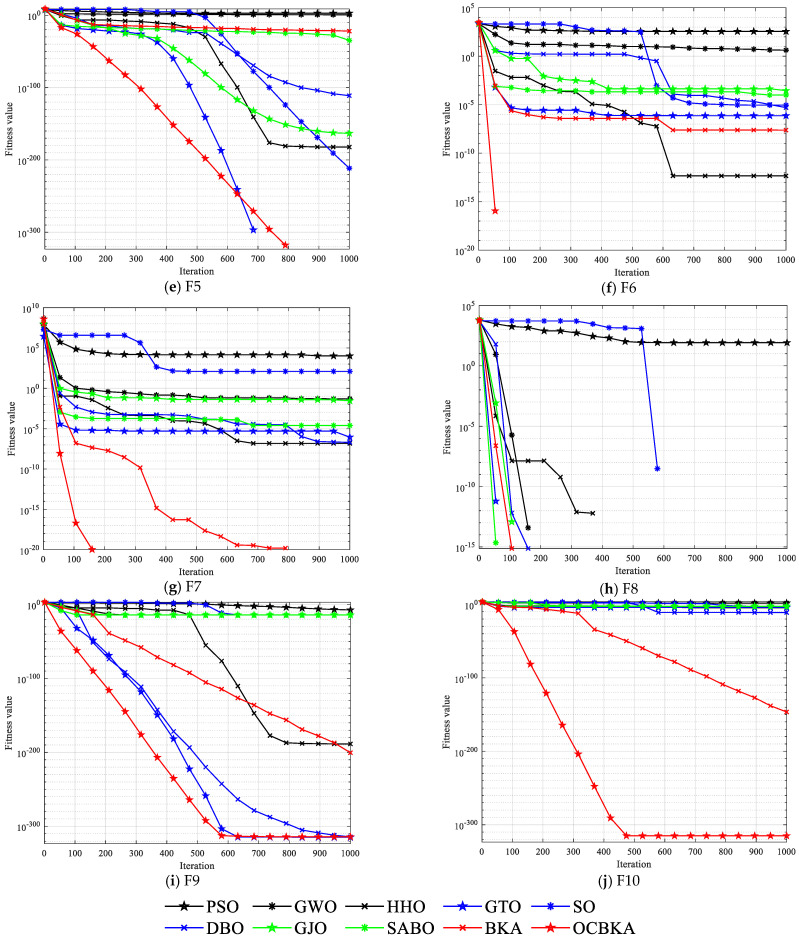
Iteration curves of different improved algorithms.

**Figure 5:**
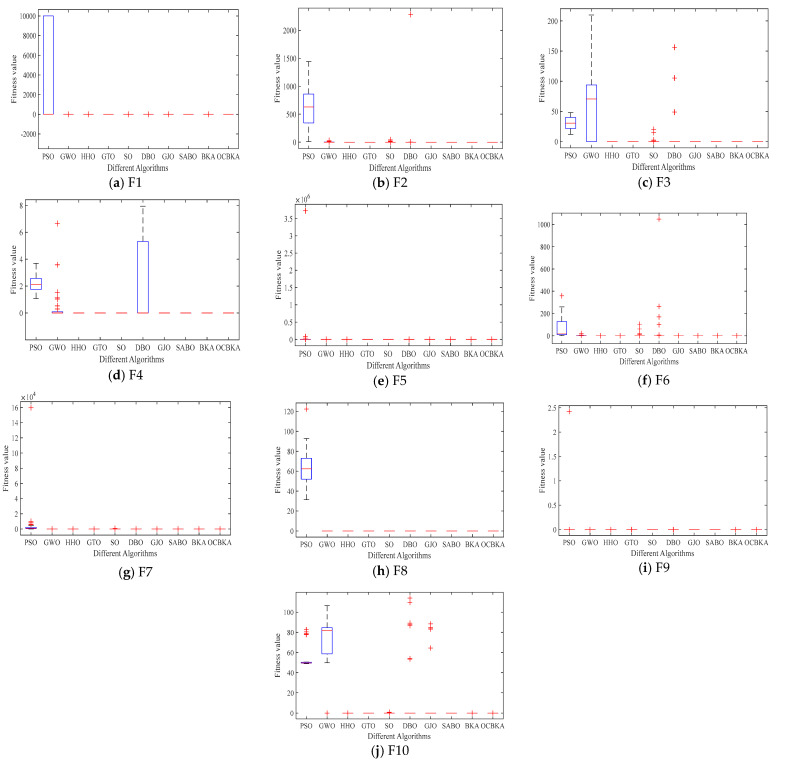
Box plots of iterative data distributions.

To confirm the viability and efficacy of the suggested enhanced algorithm in optimization problems, the CEC2021 test set was solved using OCBKA, BKA, and the remaining eight methods. Table 3 displays the test set’s detailed information. F2 to F4 are multimodal functions, F5 to F7 are mixed functions, and F8 to F10 are combined functions. Of these, F1 is a little unimodal function. The algorithm was configured as follows: the population, N = 30, the evaluation times, T = 30,000 times, and dimensions in number, D = 20. Each algorithm was executed thirty times on its own. For every test function, Table 4 shows the best solution findings’ average, standard deviation, optimal value, running time, and convergence. The best findings are indicated by bolded data. The average convergence curve of the iteration is shown in Figure 4, and the data box plot is shown in Figure 5.

Table 4 data analysis revealed that the improved algorithm in this paper solved the unimodal function F1 with the minimum value of the average value, optimal value, and standard deviation. When solving the multimodal functions F2 to F4, OCBKA obtained the ternary optimal value, demonstrating the best optimization and stability. In solving the combined functions F8 to F10 and mixed functions F5 to F7, OCBKA achieved a three-value optimum, with the exception of the F7 and F8 functions. It ranked second in terms of accuracy when solving functions, but it displayed good standard deviation in the majority of functions, further demonstrating the algorithm’s stability. We can determine that SO had the quickest running time by examining the timestamps of several algorithms. While the OCBKA somewhat lengthened the running time, its fast convergence speed and optimization accuracy demonstrated the efficacy of the modified approach. Convergence analysis showed that the method effectively reached convergence on the vast majority of functions after 1000 iterations. This suggests that the ideal solution found after a thousand iterations can satisfy the criteria for convergence accuracy when comparing algorithms.

The iterative data box graph provides a more understandable way to see the data distribution of the iterative findings. Figure 4 illustrates how the OCBKA consistently maintained a minimal quartile difference and outstanding solution accuracy. The numerical results were slanted toward the optimal solution, and outliers were less common than with other algorithms in the comparison. This is further evidence that the upgraded algorithm had a strong capacity to jump out of the local optimum solution since the algorithm seldom fell into the local optimal solution. Figure 3’s iteration curve illustrated how the improved algorithm, particularly for functions F6 to F12, maintained good convergence speed and high accuracy in the early stages of the iteration. The algorithm could also quickly converge to the vicinity of the optimal solution, demonstrating that the osprey search strategy made sure that exceptional individuals could direct the population search direction during the iteration.

### 4.4. Application of OCBKA in Engineering Optimization Problems

To confirm the feasibility of the improved approach in addressing real-world engineering optimization problems, two engineering optimization problems were solved using the nine upgraded optimization algorithms in Section 4.2 [35,36].

#### 4.4.1. Tension/Compression Spring Design Optimization Problem

Tension/compression spring optimization is the subject of engineering optimization problem 1 [36,37]. It is envisaged that the spring size that satisfies the requirements and has the least mass may be appropriately constructed, within the limitations of minimum deflection, vibration frequency, and shear stress [38]. The spring dimensions consist of a number of coils, P, a spring coil diameter, D, and a spring coil diameter, d. There are four restrictions and three variables in this issue [39]. The problem is described mathematically as follows:

Objective function:

(12) $$Variable\;x=\left[d,D,P\right];$$

(13) $$\min f(x)=(x_{3}+2)x_{2}x_{1}^{2};$$

Constraints:

(14) $$g_{1}(x)=1-\frac{x_{3}x_{2}^{2}}{71785x_{1}^{4}}\leq 0;$$

(15) $$g_{2}(x)=\frac{4x_{2}^{2}-x_{1}x_{2}}{12566(x_{2}x_{1}^{3}-x_{1}^{4})}+\frac{1}{5108x_{1}^{2}}-1\leq 0;$$

(16) $$g_{3}(x)=1-\frac{140.45x_{1}}{x_{2}^{2}x_{3}}\leq 0;$$

(17) $$g_{4}(x)=\frac{x_{1}+x_{2}}{1.5}-1\leq 0;$$

Value range:

(18) $$0.05\leq x_{1}\leq 2;$$

(19) $$0.25\leq x_{2}\leq 1.3;$$

(20) $$2\leq x_{3}\leq 15;$$

**Figure 6:**
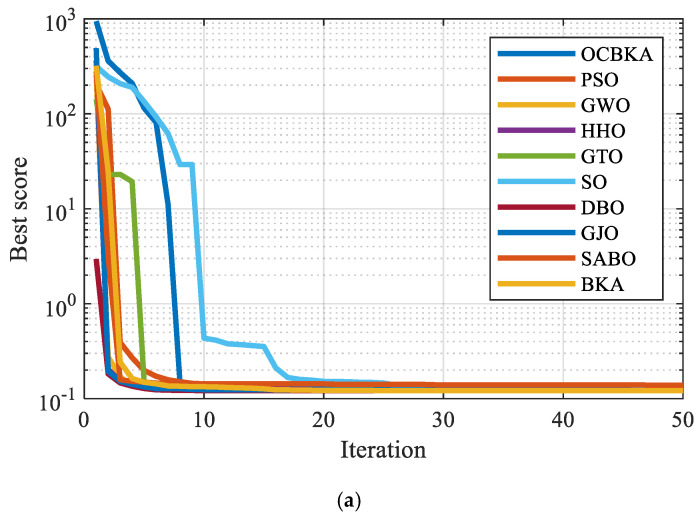

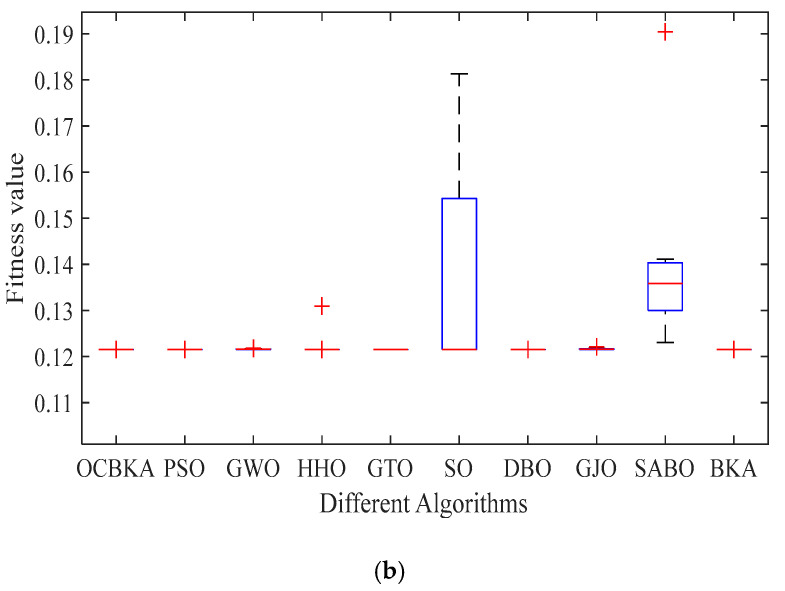
Iterative curves and box plots for tension/compression spring design. (**a**) Iterative curves of 10 algorithms. (**b**) Iterative data distribution box plot.

The engineering optimization problem 1 was solved jointly by the BKA, PSO, GWO, HHO, GTO, SO, DBO, GJO, SABO, and OCBKA algorithms, with T = 1500 evaluation times. There were thirty people in N. Ten separate runs of each method were performed, and Table 5 contains the outcomes of the solutions. Figure 6 shows the data box diagram and the average convergence curves of the iterations. Table 5’s data analysis demonstrated that, out of all the algorithms used in the computation, the OCBKA had the best mean value and convergence accuracy. This indicates that the algorithm had a strong ability to converge and operated steadily. As we can see from the box diagram and iterative graph in Figure 6, the OCBKA solved the tension/compression spring optimization issue with high stability and convergence.

#### 4.4.2. Three-Bar Truss Design Problem

A three-bar truss design optimization issue is the subject of engineering optimization problem 2 [40,41]. By resolving the cross-sectional area of the two members under stress limitations in each truss of the three-bar truss, the challenge is to build the model size with the least volume [42]. There are three restrictions and two variables in this issue. The following is the problem’s mathematical description [43]:

Objective function:

(21) $$Variable\;x=\left[x_{1},x_{2}\right];$$

(22) $$\min f(x)=(2\sqrt{2}\cdot x_{1}+x_{2})\cdot l;$$

Constraints:

(23) $$g_{1}(x)=\frac{\sqrt{2}\cdot x_{1}+x_{2}}{\sqrt{2}\cdot x_{1}^{2}+2x_{1}x_{2}}P-\sigma \leq 0;$$

(24) $$g_{2}(x)=\frac{x_{2}}{\sqrt{2}\cdot x_{1}^{2}+2x_{1}x_{2}}P-\sigma \leq 0;$$

(25) $$g_{3}(x)=\frac{1}{\sqrt{2}\cdot x_{2}+x_{1}}P-\sigma \leq 0;$$

Value range:

(26) $$0\leq x_{i}\leq 1,i=1,2;$$

(27) l=100 cm;

(28) $$P=2\;kN/cm^{2};$$

(29) $$\delta =2\;kN/cm^{2};$$

**Figure 7:**
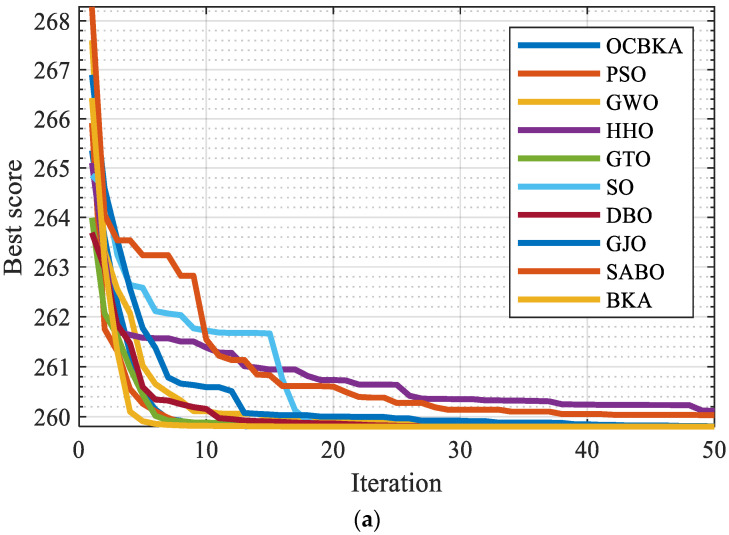

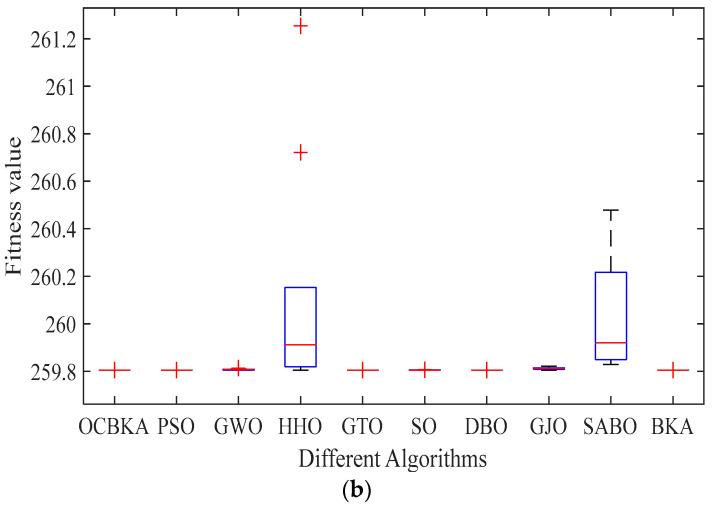
Iterative curves and box plots of the three-bar truss design optimization problem. (**a**) Iterative curves of 10 algorithms. (**b**) Iterative data distribution box plot.

To address the engineering optimization issue 2 jointly, ten algorithms—the BKA, PSO, GWO, HHO, GTO, SO, DBO, GJO, SABO, and OCBKA methods—were chosen, and the setup requirements remained the same. Table 6 contains the findings of the solution, and Figure 7 displays the data box diagram and the average convergence curves of the iteration. As we can see from the box diagram and iterative graph in Figure 7, the OCBKA solved the three-bar truss design optimization issue with high stability and convergence. Table 6’s data show that all of the algorithms used in the calculation converged to a region close to the optimal solution; of these, the OCBKA had the highest accuracy and the best average performance, demonstrating the enhanced algorithm’s effectiveness and potential utility in real-world engineering applications.

#### 4.4.3. Weight Minimization of a Speed Reducer

A common mechanical optimization problem is the reducer design problem. Its objective is to lower the reducer’s weight as much as possible to enable the engine and propeller to run normally. There are seven decision variables in the issue [44]. The problem is described mathematically as follows:

Objective function:

(30) $$\begin{array}{ll} \min f(x)= & 0.7854x_{1}x_{2}^{2}(3.3333x_{3}^{2}+14.9334x_{3}-43.0934)-1.508x_{1}(x_{6}^{3}+x_{7}^{2})\\ & +7.4777(x_{6}^{3}+x_{7}^{3})+0.7854(x_{4}x_{6}^{2}+x_{5}x_{7}^{2}) \end{array}$$

Constraints:

(31) $$g_{1}(x)=\frac{27}{x_{1}x_{2}^{2}x_{3}}-1\leq 0$$

(32) $$g_{2}(x)=\frac{397.5}{x_{1}x_{2}^{2}x_{3}}-1\leq 0$$

(33) $$g_{3}(x)=\frac{1.93x_{5}^{3}}{x_{1}x_{6}^{4}x_{3}}-1\leq 0$$

(34) $$g_{4}(x)=\frac{1.93x_{5}^{3}}{x_{2}x_{7}^{4}x_{3}}-1\leq 0$$

(35) $$g_{5}(x)=\frac{{\left[{\left(745(x_{4}/x_{2}x_{3})\right)}^{2}+16.9\times 10^{6}\right]}^{1/2}}{110x_{6}^{3}}-1\leq 0$$

(36) $$g_{6}(x)=\frac{{\left[{\left(745(x_{5}/x_{2}x_{3})\right)}^{2}+157.5\times 10^{6}\right]}^{1/2}}{85x_{7}^{3}}-1\leq 0$$

(37) $$g_{7}(x)=\frac{x_{2}x_{3}}{40}-1\leq 0$$

(38) $$g_{8}(x)=\frac{5x_{2}}{x_{1}}-1\leq 0$$

(39) $$g_{9}(x)=\frac{5x_{1}}{12x_{2}}-1\leq 0$$

(40) $$g_{10}(x)=\frac{1.5x_{6}+1.9}{x_{4}}-1\leq 0$$

(41) $$g_{11}(x)=\frac{1.1x_{7}+1.9}{x_{5}}-1\leq 0$$

Value range:

(42) $$2.6\leq x_{1}\leq 3.6;$$

(43) $$0.7\leq x_{2}\leq 0.8;$$

(44) $$17\leq x_{3}\leq 28;$$

(45) $$7.3\leq x_{4};$$

(46) $$x_{5}\leq 8.3;$$

(47) $$2.9\leq x_{6}\leq 3.9;$$

(48) $$5.0\leq x_{7}\leq 5.5;$$

**Figure 8:**
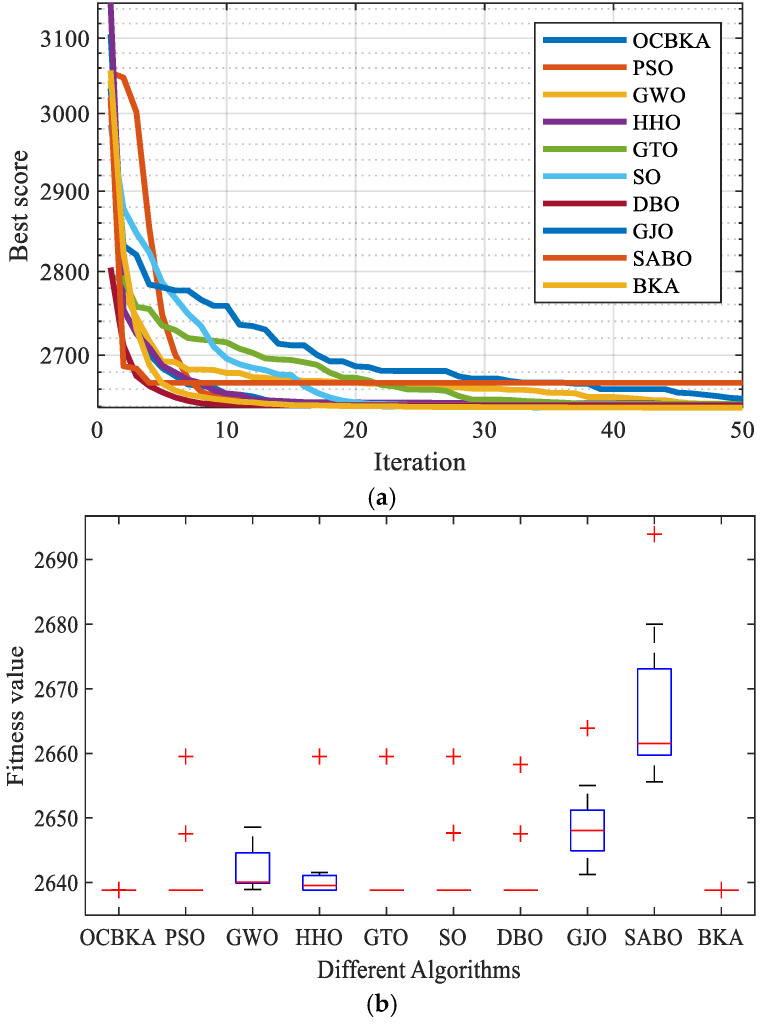
Iterative curves and box plots of the speed reducer optimization problem. (**a**) Iterative curves of 10 algorithms. (**b**) Iterative data distribution box plot.

The experimental findings for the 10 strategies used to solve the reducer design challenge are displayed in Table 7. Figure 8 displays the data box diagram and the average convergence curves of the iteration. Table 7 demonstrates that the OCBKA method yielded a minimum weight of 2638.8, indicating a high level of optimization accuracy and stability. The box diagram in Figure 8 shows that the stability of the OCBKA was superior to that of the other algorithms, suggesting that the OCBKA has superior stability and optimization capabilities.

## 5. Conclusions

In this research, a black-winged kite optimization algorithm (OCBKA) merged with osprey was produced by examining and investigating the regular BKA method. First off, the uniform distribution of people in the starting population was guaranteed by the introduction of the logistic mapping population initialization operation, which helped to improve the algorithm’s convergence speed and accuracy. The osprey optimization algorithm was then incorporated to minimize the black-winged kite’s reliance on the position of the black-winged kite from the previous generation, increase the black-winged kite’s search range in uncharted territory, optimize population diversity, and strike a balance between local development and global exploration.

The OCBKA effectively produced optimum solutions on 19 of the 23 test functions in CEC2005, according to simulation studies conducted on 10 benchmark test functions of CEC2005 and CEC2021. On seven of the ten test functions in CEC2021 that the OCBKA was able to find the best solution for, the experimental findings confirmed the better stability and enhanced optimization capabilities of the OCBKA presented in this study. Further proving the OCBKA’s efficacy and viability in real-world engineering applications, simulation experiments involving reducer engineering, three-bar truss engineering, and tension/compression spring engineering all yielded the optimal solution in all three engineering applications.

The OCBKA had improved global exploration capabilities in the early stages; however, it exhibited general stability issues in some test functions. The algorithm’s overall performance will be enhanced, and its optimization capability will be further enhanced in the future by focusing on these issues.

## Figures and Tables

**Table 1:** The test functions of CEC2005.

	Number	Function	Theoretical Value
	F1	Sphere Function	0
	F2	Schwefel’s Problem 2.22	0
Unimodal	F3	Schwefel’s Problem 1.2	0
Functions	F4	Schwefel’s Problem 2.21	0
	F5	Generalized Rosenbrock’s Function	0
	F6	Step Function	0
	F7	Quartic Function, i.e., Noise	0
	F8	Generalized Schwefel’s Problem 2.26	−12,569.5
Simple	F9	Generalized Rastrigin’s Function	0
Multimodal	F10	Ackley’s Function	0
Functions	F11	Generalized Griewank’s Function	0
	F12	Generalized Penalized Function 1	0
	F13	Generalized Penalized Function 2	0
	F14	Shekel’s Foxholes Function	0.99800383
	F15	Kowalik’s Function	0.0003075
	F16	Six-Hump Camel-Back Function	−1.03162845
	F17	Branin Function	0.39788735
Composition	F18	Goldstein–Price Function	2.99999999
Functions	F19	Hartman’s Family	−3.86278214
	F20	Hartman’s Family	−3.32199517
	F21	Shekel’s Family	−10
	F22	Shekel’s Family	−10
	F23	Shekel’s Family	−10

**Table 2:** The test results of 10 algorithms.

		PSO	GWO	HHO	GTO	SO	DBO	GJO	SABO	BKA	OCBKA
	best	1.12 × 10^−9^	2.63 × 10^−61^	5.07 × 10^−212^	**0**	3.40 × 10^−192^	**0.00 × 10^0^**	1.07 × 10^−116^	**0.00 × 10^0^**	1.36 × 10^−205^	**0.00 × 10^0^**
	std.	1.78 × 10^−6^	8.96 × 10^−59^	**0**	**0**	**0**	**0**	9.67 × 10^−110^	**0.00 × 10^0^**	8.06 × 10^−144^	**0.00 × 10^0^**
F1	avg.	6.02 × 10^−7^	4.32 × 10^−59^	1.41 × 10^−185^	**0.00 × 10^0^**	1.72 × 10^−186^	8.81 × 10^−248^	1.78 × 10^−110^	**0.00 × 10^0^**	1.47 × 10^−144^	**0.00 × 10^0^**
	time	**6.64 × 10^−2^**	1.19 × 10^−1^	1.05 × 10^−1^	2.42 × 10^−1^	6.67 × 10^−2^	1.10 × 10^−1^	1.60 × 10^−1^	1.42 × 10^−1^	1.06 × 10^−1^	1.97 × 10^−1^
	convergence	Yes	Yes	Yes	Yes	Yes	Yes	Yes	Yes	Yes	Yes
	best	6.61 × 10^−6^	9.41 × 10^−36^	1.66 × 10^−108^	**0.00 × 10^0^**	5.54 × 10^−98^	3.37 × 10^−166^	8.42 × 10^−69^	2.54 × 10^−227^	1.42 × 10^−105^	**0.00 × 10^0^**
	std.	3.45 × 10^0^	1.18 × 10^−34^	1.10 × 10^−95^	**0.00 × 10^0^**	1.86 × 10^−90^	6.24 × 10^−120^	4.68 × 10^−66^	**0.00 × 10^0^**	2.85 × 10^−84^	**0.00 × 10^0^**
F2	avg.	1.33 × 10^0^	1.19 × 10^−34^	2.03 × 10^−96^	**0.00 × 10^0^**	6.89 × 10^−91^	1.14 × 10^−120^	2.99 × 10^−66^	6.06 × 10^−223^	6.34 × 10^−85^	**0.00 × 10^0^**
	time	6.27 × 10^−2^	1.22 × 10^−1^	9.90 × 10^−2^	2.41 × 10^−1^	**5.62 × 10^−2^**	1.05 × 10^−1^	1.56 × 10^−1^	1.41 × 10^−1^	9.60 × 10^−2^	2.18 × 10^−1^
	convergence	Yes	Yes	Yes	Yes	Yes	Yes	Yes	Yes	Yes	Yes
	best	146.96 × 10^0^	2.39 × 10^−19^	3.73 × 10^−179^	**0.00 × 10^0^**	9.82 × 10^−140^	3.49 × 10^−276^	8.43 × 10^−48^	5.68 × 10^−176^	3.74 × 10^−204^	**0.00 × 10^0^**
	std.	3.24 × 10^3^	1.69 × 10^−14^	1.76 × 10^−143^	**0.00 × 10^0^**	4.52 × 10^−122^	3.84 × 10^−158^	1.03 × 10^−37^	4.90 × 10^−81^	**0.00 × 10^0^**	**0.00 × 10^0^**
F3	avg.	2.06 × 10^3^	5.72 × 10^−15^	3.98 × 10^−144^	**0.00 × 10^0^**	9.30 × 10^−123^	7.02 × 10^−159^	1.92 × 10^−38^	8.95 × 10^−82^	3.76 × 10^−179^	**0.00 × 10^0^**
	time	2.19 × 10^−1^	2.79 × 10^−1^	5.00 × 10^−1^	5.58 × 10^−1^	**2.13 × 10^−1^**	2.64 × 10^−1^	3.37 × 10^−1^	3.03 × 10^−1^	4.28 × 10^−1^	5.52 × 10^−1^
	convergence	No	Yes	Yes	Yes	Yes	Yes	Yes	Yes	Yes	Yes
	best	1.98 × 10^0^	6.31 × 10^−16^	1.70 × 10^−105^	**0.00 × 10^0^**	1.29 × 10^−89^	1.93 × 10^−157^	1.94 × 10^−36^	4.73 × 10^−158^	1.6 × 10^−103^	**0.00 × 10^0^**
	std.	1.01 × 10^0^	1.17 × 10^−14^	1.12 × 10^−91^	**0.00 × 10^0^**	6.55 × 10^−85^	7.05 × 10^−113^	4.12 × 10^−33^	8.51 × 10^−155^	2.12 × 10^−75^	**0.00 × 10^0^**
F4	avg.	3.97 × 10^0^	1.11 × 10^−14^	2.93 × 10^−92^	**0.00 × 10^0^**	3.52 × 10^−85^	1.29 × 10^−113^	1.91 × 10^−33^	4.20 × 10^−155^	3.87 × 10^−76^	**0.00 × 10^0^**
	time	6.13 × 10^−2^	1.21 × 10^−1^	1.28 × 10^−1^	2.36 × 10^−1^	**5.00 × 10^−2^**	9.81 × 10^−2^	1.53 × 10^−1^	1.42 × 10^−1^	1.04 × 10^−1^	1.97 × 10^−1^
	convergence	No	Yes	Yes	Yes	Yes	Yes	Yes	Yes	Yes	Yes
	best	2.28 × 10^1^	2.57 × 10^1^	1.02 × 10^−5^	8.4 × 10^−8^	1.82 × 10^−1^	2.45 × 10^1^	2.59 × 10^1^	2.70 × 10^1^	2.49 × 10^1^	**0.00 × 10^0^**
	std.	1.64 × 10^4^	7.33 × 10^−1^	**3.08** × 10^−3^	3.97 × 10^0^	1.25 × 10^1^	1.73 × 10^−1^	9.54 × 10^−1^	6.12 × 10^−1^	1.22 × 10^0^	1.31 × 10^1^
F5	avg.	3.39 × 10^3^	2.70 × 10^1^	**0.26** × 10^−3^	7.26 × 10^−1^	1.39 × 10^1^	2.49 × 10^1^	2.76 × 10^1^	2.80 × 10^1^	2.71 × 10^1^	1.38 × 10^1^
	time	8.23 × 10^−2^	1.35 × 10^−1^	1.99 × 10^−1^	2.56 × 10^−1^	**7.03 × 10^−2^**	1.20 × 10^−1^	1.74 × 10^−1^	1.57 × 10^−1^	1.43 × 10^−1^	2.40 × 10^−1^
	convergence	No	Yes	Yes	Yes	Yes	No	Yes	Yes	Yes	Yes
	best	6.34 × 10^−10^	2.1 × 10^−5^	4.77 × 10^−8^	4.61 × 10^−16^	9.27 × 10^−7^	7.24 × 10^−11^	1.75 × 10^0^	1.22 × 10^0^	7.26 × 10^−5^	**0.00 × 10^0^**
	std.	1.72 × 10^−5^	4.17 × 10^−1^	4.30 × 10^−5^	8.08 × 10^−12^	2.73 × 10^0^	2.01 × 10^−7^	3.77 × 10^−1^	6.01 × 10^−1^	1.58 × 10^0^	**1.24 × 10^−12^**
F6	avg.	3.27 × 10^−6^	7.35 × 10^−1^	2.63 × 10^−5^	3.83 × 10^−12^	2.05 × 10^0^	5.75 × 10^−8^	2.59 × 10^0^	2.04 × 10^0^	9.91 × 10^−1^	**2.29 × 10^−13^**
	time	5.96 × 10^−2^	1.17 × 10^−1^	1.46 × 10^−1^	2.26 × 10^−1^	**5.18 × 10^−2^**	9.88 × 10^−2^	1.52 × 10^−1^	1.38 × 10^−1^	1.04 × 10^−1^	1.91 × 10^−1^
	convergence	Yes	Yes	Yes	Yes	Yes	Yes	Yes	Yes	Yes	Yes
	best	1.27 × 10^−2^	2.66 × 10^−4^	2.32 × 10^−6^	**7.9 × 10^−7^**	9.6 × 10^−7^	3.56 × 10^−5^	3.41 × 10^−5^	3.25 × 10^−6^	6.60 × 10^−6^	2.97 × 10^−6^
	std.	1.00 × 10^−2^	3.98 × 10^−4^	9.28 × 10^−5^	4.45 × 10^−5^	7.96 × 10^−5^	5.14 × 10^−4^	2.57 × 10^−4^	4.78 × 10^−5^	7.12 × 10^−5^	**3.61 × 10^−5^**
F7	avg.	2.76 × 10^−2^	7.72 × 10^−4^	9.67 × 10^−5^	4.92 × 10^−5^	9.3 × 10^−5^	5.65 × 10^−4^	2.44 × 10^−4^	5.42 × 10^−5^	1.18 × 10^−4^	**4.58 × 10^−5^**
	time	1.66 × 10^−1^	2.21 × 10^−1^	3.61 × 10^−1^	4.30 × 10^−1^	**1.56 × 10^−1^**	2.03 × 10^−1^	2.65 × 10^−1^	2.42 × 10^−1^	3.14 × 10^−1^	4.14 × 10^−1^
	convergence	Yes	Yes	Yes	Yes	Yes	Yes	Yes	Yes	Yes	Yes
	best	−9.78 × 10^3^	−7.15 × 10^3^	**−1.26 × 10^4^**	**−1.26 × 10^4^**	**−1.26 × 10^4^**	−1.25 × 10^4^	−6.50 × 10^3^	−4.59 × 10^3^	−1.13 × 10^4^	−1.26 × 10^4^
	std.	5.70 × 10^2^	7.67 × 10^2^	1.19 × 10^2^	**2.29 × 10^−9^**	4.51 × 10^1^	1.81 × 10^3^	1.03 × 10^3^	5.36 × 10^2^	1.89 × 10^3^	4.75 × 10^1^
F8	avg.	−8.78 × 10^3^	−6.04 × 10^3^	−1.25 × 10^4^	**−1.26 × 10^4^**	−1.25 × 10^4^	−8.93 × 10^3^	−3.82 × 10^3^	−3.20 × 10^3^	−8.85 × 10^3^	−1.26 × 10^4^
	time	8.52 × 10^−2^	1.43 × 10^−1^	2.09 × 10^−1^	2.77 × 10^−1^	**7.30 × 10^−2^**	1.37 × 10^−1^	1.81 × 10^−1^	1.62 × 10^−1^	1.49 × 10^−1^	2.44 × 10^−1^
	convergence	Yes	No	Yes	Yes	Yes	No	No	Yes	No	No
	best	2.98 × 10^1^	0.00 × 10^0^	**0.00 × 10^0^**	**0.00 × 10^0^**	**0.00 × 10^0^**	**0.00 × 10^0^**	**0.00 × 10^0^**	**0.00 × 10^0^**	**0.00 × 10^0^**	**0.00 × 10^0^**
	std.	1.72 × 10^1^	1.00 × 10^0^	**0.00 × 10^0^**	**0.00 × 10^0^**	5.49 × 10^0^	2.91 × 10^0^	**0.00 × 10^0^**	**0.00 × 10^0^**	**0.00 × 10^0^**	**0.00 × 10^0^**
F9	avg.	5.82 × 10^1^	3.28 × 10^−1^	**0.00 × 10^0^**	**0.00 × 10^0^**	1.00 × 10^0^	5.31 × 10^−1^	**0.00 × 10^0^**	**0.00 × 10^0^**	**0.00 × 10^0^**	**0.00 × 10^0^**
	time	7.81 × 10^−2^	1.19 × 10^−1^	1.69 × 10^−1^	2.39 × 10^−1^	**6.56 × 10^−2^**	1.05 × 10^−1^	1.59 × 10^−1^	1.41 × 10^−1^	1.13 × 10^−1^	1.96 × 10^−1^
	convergence	Yes	Yes	Yes	Yes	Yes	Yes	Yes	Yes	Yes	Yes
	best	7.31 × 10^−6^	1.51 × 10^−14^	**8.88 × 10^−16^**	**8.88 × 10^−16^**	**8.88 × 10^−16^**	**8.88 × 10^−16^**	4.44 × 10^−15^	4.44 × 10^−15^	**8.88 × 10^−16^**	**8.88 × 10^−16^**
	std.	5.36 × 10^−1^	2.75 × 10^−15^	**0.00 × 10^0^**	**0.00 × 10^0^**	6.49 × 10^−16^	**0.00 × 10^0^**	9.01 × 10^−16^	6.49 × 10^−16^	**0.00 × 10^0^**	**0.00 × 10^0^**
F10	avg.	2.55 × 10^−1^	1.66 × 10^−14^	**8.88 × 10^−16^**	**8.88 × 10^−16^**	4.32 × 10^−15^	**8.88 × 10^−16^**	4.68 × 10^−15^	4.56 × 10^−15^	**8.88 × 10^−16^**	**8.88 × 10^−16^**
	time	8.20 × 10^−2^	1.22 × 10^−1^	1.78 × 10^−1^	2.37 × 10^−1^	**6.44 × 10^−2^**	1.08 × 10^−1^	1.60 × 10^−1^	1.45 × 10^−1^	9.94 × 10^−2^	2.00 × 10^−1^
	convergence	Yes	Yes	Yes	Yes	Yes	Yes	Yes	Yes	Yes	Yes
	best	1.83 × 10^−8^	**0.00 × 10^0^**	**0.00 × 10^0^**	**0.00 × 10^0^**	**0.00 × 10^0^**	**0.00 × 10^0^**	**0.00 × 10^0^**	**0.00 × 10^0^**	**0.00 × 10^0^**	**0.00 × 10^0^**
	std.	1.83 × 10^−8^	0.00 × 10^0^	**0.00 × 10^0^**	**0.00 × 10^0^**	**0.00 × 10^0^**	**0.00 × 10^0^**	**0.00 × 10^0^**	**0.00 × 10^0^**	**0.00 × 10^0^**	**0.00 × 10^0^**
F11	avg.	1.72 × 10^−2^	5.34 × 10^−3^	**0.00 × 10^0^**	**0.00 × 10^0^**	**0.00 × 10^0^**	**0.00 × 10^0^**	**0.00 × 10^0^**	**0.00 × 10^0^**	**0.00 × 10^0^**	**0.00 × 10^0^**
	time	9.27 × 10^−2^	1.40 × 10^−1^	2.08 × 10^−1^	2.62 × 10^−1^	**7.47 × 10^−2^**	1.25 × 10^−1^	1.76 × 10^−1^	1.66 × 10^−1^	1.50 × 10^−1^	2.30 × 10^−1^
	convergence	Yes	Yes	Yes	Yes	Yes	Yes	Yes	Yes	Yes	Yes
	best	4.32 × 10^−11^	1.31 × 10^−2^	1.05 × 10^−9^	1.43 × 10^−14^	1.82 × 10^−6^	6.68 × 10^−13^	1.70 × 10^−1^	5.64 × 10^−2^	8.90 × 10^−6^	**1.57 × 10^−32^**
	std.	1.58 × 10^−1^	1.95 × 10^−2^	2.72 × 10^−6^	1.78 × 10^−12^	8.88 × 10^−3^	8.54 × 10^−4^	4.97 × 10^−2^	1.44 × 10^−1^	6.96 × 10^−2^	**9.92 × 10^−34^**
F12	avg.	8.31 × 10^−2^	3.97 × 10^−2^	2.38 × 10^−6^	7.81 × 10^−13^	8.97 × 10^−3^	1.56 × 10^−4^	2.19 × 10^−1^	1.57 × 10^−1^	3.35 × 10^−2^	**1.60 × 10^−32^**
	time	3.38 × 10^−1^	3.86 × 10^−1^	8.11 × 10^−1^	7.68 × 10^−1^	**3.25 × 10^−1^**	3.79 × 10^−1^	4.80 × 10^−1^	4.15 × 10^−1^	6.54 × 10^−1^	7.38 × 10^−1^
	convergence	Yes	Yes	Yes	Yes	Yes	Yes	Yes	Yes	Yes	Yes
	best	2.38 × 10^−9^	0.197789	4.01 × 10^−8^	**2.78 × 10^−14^**	9.24 × 10^−5^	5.87 × 10^−7^	1.156906	1.343081	0.554653	**1.35 × 10^−32^**
	std.	1.10 × 10^−2^	2.05 × 10^−1^	3.58 × 10^−5^	**1.02 × 10^−2^**	5.88 × 10^−2^	2.92 × 10^−1^	2.67 × 10^−1^	5.39 × 10^−1^	5.56 × 10^−1^	2.63 × 10^−2^
F13	avg.	6.61 × 10^−3^	5.02 × 10^−1^	**2.21 × 10^−5^**	2.56 × 10^−3^	6.19 × 10^−2^	3.14 × 10^−1^	1.68 × 10^0^	2.67 × 10^0^	1.60 × 10^0^	5.84 × 10^−3^
	time	3.40 × 10^−1^	3.92 × 10^−1^	8.09 × 10^−1^	7.77 × 10^−1^	**3.26 × 10^−1^**	3.75 × 10^−1^	4.77 × 10^−1^	4.17 × 10^−1^	6.57 × 10^−1^	7.45 × 10^−1^
	convergence	Yes	Yes	Yes	Yes	Yes	Yes	Yes	Yes	Yes	Yes
	best	**9.98 × 10^−1^**	**9.98 × 10^−1^**	**9.98 × 10^−1^**	**9.98 × 10^−1^**	**9.98 × 10^−1^**	**9.98 × 10^−1^**	**9.98 × 10^−1^**	**9.98 × 10^−1^**	**9.98 × 10^−1^**	**9.98 × 10^−1^**
	std.	0.00 × 10^0^	4.58 × 10^0^	3.03 × 10^−1^	**0.00 × 10^0^**	4.26 × 10^−7^	1.88 × 10^0^	4.35 × 10^0^	2.87 × 10^0^	7.38 × 10^−1^	1.02 × 10^0^
F14	avg.	**9.98 × 10^−1^**	6.44 × 10^0^	1.10 × 10^0^	**9.98 × 10^−1^**	**9.98 × 10^−1^**	1.62 × 10^0^	6.09 × 10^0^	3.11 × 10^0^	1.16 × 10^0^	1.63 × 10^0^
	time	**5.05 × 10^−1^**	5.07 × 10^−1^	1.30 × 10^0^	1.08 × 10^0^	5.10 × 10^−1^	5.62 × 10^−1^	5.59 × 10^−1^	5.46 × 10^−1^	1.03 × 10^0^	1.11 × 10^0^
	convergence	Yes	Yes	Yes	Yes	Yes	Yes	Yes	Yes	Yes	Yes
	best	**3.07 × 10^−4^**	**3.07 × 10^−4^**	3.08 × 10^−4^	**3.07 × 10^−4^**	3.08 × 10^−4^	**3.07 × 10^−4^**	**3.07 × 10^−4^**	3.18 × 10^−4^	**3.07 × 10^−4^**	**3.07 × 10^−4^**
	std.	6.07 × 10^−3^	7.58 × 10^−3^	1.66 × 10^−4^	2.79 × 10^−4^	3.02 × 10^−4^	3.01 × 10^−4^	3.17 × 10^−4^	3.84 × 10^−3^	3.78 × 10^−4^	**6.51 × 10^−9^**
F15	avg.	2.48 × 10^−3^	3.72 × 10^−3^	3.62 × 10^−4^	3.99 × 10^−4^	6.26 × 10^−4^	6.12 × 10^−4^	4.22 × 10^−4^	1.24 × 10^−3^	4.80 × 10^−4^	**3.07 × 10^−4^**
	time	**3.73 × 10^−2^**	4.44 × 10^−2^	1.21 × 10^−1^	1.54 × 10^−1^	3.93 × 10^−2^	9.57 × 10^−2^	9.21 × 10^−2^	7.88 × 10^−2^	9.27 × 10^−2^	1.73 × 10^−1^
	convergence	Yes	Yes	Yes	Yes	Yes	Yes	Yes	Yes	Yes	Yes
	best	**−1.03 × 10^0^**	**−1.03 × 10^0^**	**−1.03 × 10^0^**	**−1.03 × 10^0^**	**−1.03 × 10^0^**	**−1.03 × 10^0^**	**−1.03 × 10^0^**	**−1.03 × 10^0^**	**−1.03 × 10^0^**	**−1.03 × 10^0^**
	std.	6.71 × 10^−16^	5.67 × 10^−9^	3.38 × 10^−11^	6.71 × 10^−16^	1.49 × 10^−1^	6.25 × 10^−16^	4.21 × 10^−8^	1.23 × 10^−2^	6.12 × 10^−16^	6.25 × 10^−16^
F16	avg.	**−1.03 × 10^0^**	**−1.03 × 10^0^**	**−1.03 × 10^0^**	**−1.03 × 10^0^**	−1.00 × 10^0^	**−1.03 × 10^0^**	**−1.03 × 10^0^**	**−1.03 × 10^0^**	**−1.03 × 10^0^**	**−1.03 × 10^0^**
	time	3.95 × 10^−2^	4.17 × 10^−2^	1.27 × 10^−1^	1.54 × 10^−1^	**3.87 × 10^−2^**	9.36 × 10^−2^	8.64 × 10^−2^	7.58 × 10^−2^	9.00 × 10^−2^	1.60 × 10^−1^
	convergence	Yes	Yes	Yes	Yes	Yes	Yes	Yes	Yes	Yes	Yes
	best	3.98 × 10^−1^	3.98 × 10^−1^	3.98 × 10^−1^	3.98 × 10^−1^	3.98 × 10^−1^	3.98 × 10^−1^	3.98 × 10^−1^	3.98 × 10^−1^	3.98 × 10^−1^	3.98 × 10^−1^
	std.	**0.00 × 10^0^**	7.51 × 10^−7^	4.56 × 10^−7^	**0.00 × 10^0^**	**0.00 × 10^0^**	**0.00 × 10^0^**	1.48 × 10^−5^	2.34 × 10^−1^	1.27 × 10^−9^	**0.00 × 10^0^**
F17	avg.	**3.98 × 10^−1^**	**3.98 × 10^−1^**	**3.98 × 10^−1^**	**3.98 × 10^−1^**	**3.98 × 10^−1^**	**3.98 × 10^−1^**	**3.98 × 10^−1^**	5.10 × 10^−1^	**3.98 × 10^−1^**	**3.98 × 10^−1^**
	time	**3.02 × 10^−2^**	3.30 × 10^−2^	1.05 × 10^−1^	1.46 × 10^−1^	3.86 × 10^−2^	9.06 × 10^−2^	1.07 × 10^−1^	1.07 × 10^−1^	7.51 × 10^−2^	1.38 × 10^−1^
	convergence	Yes	Yes	Yes	Yes	Yes	Yes	Yes	Yes	Yes	Yes
	best	**3.00 × 10^0^**	**3.00 × 10^0^**	**3.00 × 10^0^**	**3.00 × 10^0^**	**3.00 × 10^0^**	**3.00 × 10^0^**	**3.00 × 10^0^**	**3.00 × 10^0^**	**3.00 × 10^0^**	**3.00 × 10^0^**
	std.	1.38 × 10^−15^	1.48 × 10^1^	3.80 × 10^−8^	1.41 × 10^−15^	1.12 × 10^1^	2.15 × 10^−15^	7.51 × 10^−7^	2.61 × 10^0^	1.31 × 10^−15^	**7.56 × 10^−16^**
F18	avg.	**3.00 × 10^0^**	5.70 × 10^0^	**3.00 × 10^0^**	**3.00 × 10^0^**	8.49 × 10^0^	**3.00 × 10^0^**	**3.00 × 10^0^**	4.28 × 10^0^	**3.00 × 10^0^**	**3.00 × 10^0^**
	time	**2.57 × 10^−2^**	3.16 × 10^−2^	9.91 × 10^−2^	1.37 × 10^−1^	3.14 × 10^−2^	9.91 × 10^−2^	7.78 × 10^−2^	6.30 × 10^−2^	7.90 × 10^−2^	1.39 × 10^−1^
	convergence	Yes	Yes	Yes	Yes	Yes	Yes	Yes	Yes	Yes	Yes
	best	**−3.86 × 10^0^**	**−3.86 × 10^0^**	**−3.86 × 10^0^**	**−3.86 × 10^0^**	**−3.86 × 10^0^**	**−3.86 × 10^0^**	**−3.86 × 10^0^**	**−3.86 × 10^0^**	**−3.86 × 10^0^**	**−3.86 × 10^0^**
	std.	2.68 × 10^−15^	2.35 × 10^−3^	1.41 × 10^−3^	2.67 × 10^−15^	1.41 × 10^−1^	2.40 × 10^−3^	3.89 × 10^−3^	2.06 × 10^−1^	2.50 × 10^−15^	3.44 × 10^−8^
F19	avg.	**−3.86 × 10^0^**	**−3.86 × 10^0^**	**−3.86 × 10^0^**	**−3.86 × 10^0^**	−3.84 × 10^0^	**−3.86 × 10^0^**	**−3.86 × 10^0^**	−3.62 × 10^0^	**−3.86 × 10^0^**	**−3.86 × 10^0^**
	time	**4.21 × 10^−2^**	4.75 × 10^−2^	1.39 × 10^−1^	1.76 × 10^−1^	4.66 × 10^−2^	9.99 × 10^−2^	9.32 × 10^−2^	8.24 × 10^−2^	1.02 × 10^−1^	1.71 × 10^−1^
	convergence	Yes	Yes	Yes	Yes	Yes	Yes	Yes	Yes	Yes	Yes
	best	−3.32 × 10^0^	−3.32 × 10^0^	−3.30 × 10^0^	−3.32 × 10^0^	−3.32 × 10^0^	−3.32 × 10^0^	−3.32 × 10^0^	−3.32 × 10^0^	−3.32 × 10^0^	**−3.32 × 10^0^**
	std.	9.92 × 10^−2^	6.60 × 10^−2^	8.40 × 10^−2^	5.35 × 10^−2^	7.13 × 10^−2^	1.24 × 10^−1^	2.83 × 10^−1^	9.30 × 10^−2^	7.25 × 10^−2^	**4.15 × 10^−2^**
F20	avg.	−3.26 × 10^0^	−3.28 × 10^0^	−3.18 × 10^0^	−3.29 × 10^0^	−3.26 × 10^0^	−3.18 × 10^0^	−3.07 × 10^0^	−3.25 × 10^0^	−3.28 × 10^0^	**−3.31 × 10^0^**
	time	4.81 × 10^−2^	5.55 × 10^−2^	1.46 × 10^−1^	1.71 × 10^−1^	**4.78 × 10^−2^**	1.01 × 10^−1^	1.09 × 10^−1^	9.28 × 10^−2^	1.07 × 10^−1^	1.88 × 10^−1^
	convergence	Yes	Yes	Yes	Yes	Yes	Yes	Yes	Yes	Yes	Yes
	best	−1.02 × 10^1^	−1.02 × 10^1^	**−1.01 × 10^1^**	−1.02 × 10^1^	−1.02 × 10^1^	−1.02 × 10^1^	−1.02 × 10^1^	−8.22 × 10^0^	−1.02 × 10^1^	−1.02 × 10^1^
	std.	3.57 × 10^0^	2.06 × 10^0^	9.23 × 10^−1^	**6.68 × 10^−15^**	1.99 × 10^−1^	2.36 × 10^0^	2.68 × 10^0^	9.03 × 10^−1^	2.18 × 10^−7^	1.30 × 10^−7^
F21	avg.	−6.24 × 10^0^	−9.14 × 10^0^	−5.22 × 10^0^	−1.02 × 10^1^	**−1.01 × 10^1^**	−6.60 × 10^0^	−7.87 × 10^0^	−5.13 × 10^0^	−1.02 × 10^1^	−1.02 × 10^1^
	time	5.76 × 10^−2^	6.06 × 10^−2^	1.73 × 10^−1^	1.85 × 10^−1^	**5.49 × 10^−2^**	1.11 × 10^−1^	1.09 × 10^−1^	9.60 × 10^−2^	1.22 × 10^−1^	2.01 × 10^−1^
	convergence	Yes	Yes	Yes	Yes	Yes	Yes	Yes	Yes	Yes	Yes
	best	**−1.04 × 10^1^**	**−1.04 × 10^1^**	**−1.04 × 10^1^**	**−1.04 × 10^1^**	**−1.04 × 10^1^**	**−1.04 × 10^1^**	**−1.04 × 10^1^**	−8.89 × 10^0^	**−1.04 × 10^1^**	**−1.04 × 10^1^**
	std.	3.57 × 10^0^	9.70 × 10^−1^	9.67 × 10^−1^	**1.09 × 10^−15^**	1.63 × 10^−1^	2.89 × 10^0^	2.14 × 10^0^	1.02 × 10^0^	1.98 × 10^0^	3.97 × 10^−6^
F22	avg.	−7.30 × 10^0^	**−1.02 × 10^1^**	−5.26 × 10^0^	−1.04 × 10^1^	−1.04 × 10^1^	−8.13 × 10^0^	−9.46 × 10^0^	−5.29 × 10^0^	−9.75 × 10^0^	−1.04 × 10^1^
	time	**6.28 × 10^−2^**	6.69 × 10^−2^	1.81 × 10^−1^	2.00 × 10^−1^	6.33 × 10^−2^	1.23 × 10^−1^	1.23 × 10^−1^	1.08 × 10^−1^	1.40 × 10^−1^	2.38 × 10^−1^
	convergence	Yes	Yes	Yes	Yes	Yes	Yes	No	Yes	Yes	Yes
	best	−1.05 × 10^1^	−1.05 × 10^1^	−1.04 × 10^1^	−1.05 × 10^1^	−1.05 × 10^1^	−1.05 × 10^1^	−1.05 × 10^1^	**−9.94 × 10^0^**	−1.05 × 10^1^	−1.05 × 10^1^
	std.	3.63 × 10^0^	1.48 × 10^0^	1.35 × 10^0^	**8.73 × 10^−16^**	2.42 × 10^−2^	2.71 × 10^0^	2.14 × 10^0^	9.64 × 10^−1^	1.41 × 10^0^	8.65 × 10^−6^
F23	avg.	−8.05 × 10^0^	**−1.03 × 10^1^**	−5.48 × 10^0^	−1.05 × 10^1^	−1.05 × 10^1^	−8.20 × 10^0^	−9.72 × 10^0^	−5.27 × 10^0^	−1.03 × 10^1^	−1.05 × 10^1^
	time	**7.34 × 10^−2^**	8.07 × 10^−2^	2.18 × 10^−1^	2.24 × 10^−1^	7.70 × 10^−2^	1.36 × 10^−1^	1.32 × 10^−1^	1.17 × 10^−1^	1.66 × 10^−1^	2.41 × 10^−1^
	convergence	Yes	Yes	Yes	Yes	No	Yes	No	Yes	Yes	Yes

**Table 3:** CEC2021 partial test functions.

	No.	Functions	F_i_
Unimodal Function	1	Shifted and Rotated Bent Cigar Function	100
	2	Shifted and Rotated Schwefel’s Function	1100
Multimodal Functions	3	Shifted and Rotated Lunacek bi-Rastrigin Function	700
	4	Expanded Rosenbrock’s plus Griewangk’s Function	1900
	5	Hybrid Function 1 (N = 3)	1700
Hybrid Functions	6	Hybrid Function 2 (N = 4)	1600
	7	Hybrid Function 3 (N = 5)	2100
	8	Composition Function 1 (N = 3)	2200
Composition Functions	9	Composition Function 2 (N = 4)	2400
	10	Composition Function 3 (N = 5)	2500

**Table 4:** Test results of the algorithms improved by different strategies.

		PSO	GWO	HHO	GTO	SO	DBO	GJO	SABO	BKA	OCBKA
	best	4.47 × 10^−12^	1.32 × 10^−72^	2.26 × 10^−205^	**0.00 × 10^0^**	3.66 × 10^−195^	6.37 × 10^−295^	3.89 × 10^−144^	**0.00 × 10^0^**	9.14 × 10^−201^	**0.00 × 10^0^**
	std.	4.98 × 10^3^	1.08 × 10^−69^	**0.00 × 10^0^**	**0.00 × 10^0^**	**0.00 × 10^0^**	**0.00 × 10^0^**	5.45 × 10^−136^	**0.00 × 10^0^**	**0.00 × 10^0^**	**0.00 × 10^0^**
F1	avg.	4.00 × 10^3^	5.46 × 10^−70^	4.93 × 10^−172^	**0.00 × 10^0^**	2.67 × 10^−189^	4.24 × 10^−216^	1.34 × 10^−136^	**0.00 × 10^0^**	6.35 × 10^−175^	**0.00 × 10^0^**
	time	6.55 × 10^−2^	1.09 × 10^−1^	9.96 × 10^−2^	2.43 × 10^−1^	**5.95 × 10^−2^**	1.09 × 10^−1^	1.71 × 10^−1^	1.40 × 10^−1^	1.10 × 10^−1^	2.02 × 10^−1^
	convergence	Yes	Yes	Yes	Yes	Yes	Yes	Yes	Yes	Yes	Yes
	best	6.86 × 10^0^	**0.00 × 10^0^**	**0.00 × 10^0^**	**0.00 × 10^0^**	**0.00 × 10^0^**	**0.00 × 10^0^**	**0.00 × 10^0^**	**0.00 × 10^0^**	**0.00 × 10^0^**	**0.00 × 10^0^**
	std.	3.92 × 10^2^	8.19 × 10^0^	**0.00 × 10^0^**	**0.00 × 10^0^**	6.18 × 10^0^	8.05 × 10^2^	**0.00 × 10^0^**	8.48 × 10^−13^	**0.00 × 10^0^**	**0.00 × 10^0^**
F2	avg.	6.04 × 10^2^	2.98 × 10^0^	**0.00 × 10^0^**	**0.00 × 10^0^**	1.37 × 10^0^	3.27 × 10^2^	**0.00 × 10^0^**	5.46 × 10^−13^	**0.00 × 10^0^**	**0.00 × 10^0^**
	time	6.31 × 10^−2^	1.13 × 10^−1^	9.89 × 10^−2^	2.48 × 10^−1^	**5.74 × 10^−2^**	1.06 × 10^−1^	1.55 × 10^−1^	1.42 × 10^−1^	1.18 × 10^−1^	2.12 × 10^−1^
	convergence	Yes	Yes	Yes	Yes	Yes	Yes	Yes	Yes	Yes	Yes
	best	1.59 × 10^1^	**0.00 × 10^0^**	**0.00 × 10^0^**	**0.00 × 10^0^**	**0.00 × 10^0^**	**0.00 × 10^0^**	**0.00 × 10^0^**	**0.00 × 10^0^**	**0.00 × 10^0^**	**0.00 × 10^0^**
	std.	1.04 × 10^1^	6.26 × 10^1^	**0.00 × 10^0^**	**0.00 × 10^0^**	7.00 × 10^0^	4.28 × 10^1^	**0.00 × 10^0^**	**0.00 × 10^0^**	**0.00 × 10^0^**	**0.00 × 10^0^**
F3	avg.	3.25 × 10^1^	6.14 × 10^1^	**0.00 × 10^0^**	**0.00 × 10^0^**	2.91 × 10^0^	1.66 × 10^1^	**0.00 × 10^0^**	**0.00 × 10^0^**	**0.00 × 10^0^**	**0.00 × 10^0^**
	time	2.23 × 10^−1^	2.70 × 10^−1^	5.01 × 10^−1^	5.72 × 10^−1^	**2.17 × 10^−1^**	2.73 × 10^−1^	3.31 × 10^−1^	3.01 × 10^−1^	4.27 × 10^−1^	5.41 × 10^−1^
	convergence	No	Yes	Yes	Yes	Yes	Yes	Yes	Yes	Yes	Yes
	best	1.29 × 10^0^	**0.00 × 10^0^**	**0.00 × 10^0^**	**0.00 × 10^0^**	**0.00 × 10^0^**	**0.00 × 10^0^**	**0.00 × 10^0^**	**0.00 × 10^0^**	**0.00 × 10^0^**	**0.00 × 10^0^**
	std.	6.30 × 10^−1^	5.35 × 10^−1^	**0.00 × 10^0^**	**0.00 × 10^0^**	3.83 × 10^−2^	3.74 × 10^0^	**0.00 × 10^0^**	**0.00 × 10^0^**	**0.00 × 10^0^**	**0.00 × 10^0^**
F4	avg.	2.16 × 10^0^	2.50 × 10^−1^	**0.00 × 10^0^**	**0.00 × 10^0^**	7.00 × 10^−3^	1.80 × 10^0^	**0.00 × 10^0^**	**0.00 × 10^0^**	**0.00 × 10^0^**	**0.00 × 10^0^**
	time	5.95 × 10^−2^	1.08 × 10^−1^	1.25 × 10^−1^	2.36 × 10^−1^	**4.82 × 10^−2^**	9.83 × 10^−2^	1.53 × 10^−1^	1.39 × 10^−1^	1.05 × 10^−1^	2.00 × 10^−1^
	convergence	No	Yes	Yes	Yes	Yes	Yes	Yes	Yes	Yes	Yes
	best	4.63 × 10^1^	3.38 × 10^−28^	5.12 × 10^−204^	**0.00 × 10^0^**	2.58 × 10^−196^	1.76 × 10^−281^	6.98 × 10^−196^	3.67 × 10^−149^	4.78 × 10^−104^	**0.00 × 10^0^**
	std.	9.53 × 10^4^	3.77 × 10^0^	**0.00 × 10^0^**	**0.00 × 10^0^**	4.09 × 10^2^	4.50 × 10^1^	9.06 × 10^−127^	1.60 × 10^−26^	1.34 × 10^−24^	**0.00 × 10^0^**
F5	avg.	2.46 × 10^4^	1.83 × 10^0^	6.49 × 10^−166^	**0.00 × 10^0^**	1.87 × 10^2^	8.71 × 10^0^	1.65 × 10^−127^	5.73 × 10^−27^	2.48 × 10^−25^	1.45 × 10^−288^
	time	7.99 × 10^−2^	1.28 × 10^−1^	1.95 × 10^−1^	2.57 × 10^−1^	**7.14 × 10^−2^**	1.23 × 10^−1^	1.76 × 10^−1^	1.59 × 10^−1^	1.43 × 10^−1^	2.42 × 10^−1^
	convergence	No	Yes	Yes	Yes	Yes	No	Yes	Yes	Yes	Yes
	best	2.15 × 10^0^	3.52 × 10^−2^	−2.22 × 10^−16^	5.15 × 10^−13^	−8.51 × 10^−17^	**0.00 × 10^0^**	3.19 × 10^−5^	4.34 × 10^−6^	−2.22 × 10^−16^	−2.22 × 10^−16^
	std.	8.36 × 10^1^	2.45 × 10^0^	2.18 × 10^−4^	3.72 × 10^−6^	1.79 × 10^1^	1.53 × 10^2^	5.30 × 10^−2^	8.94 × 10^−5^	2.35 × 10^−7^	**1.29 × 10^−8^**
F6	avg.	5.89 × 10^1^	1.66 × 10^0^	5.75 × 10^−5^	3.19 × 10^−6^	4.95 × 10^0^	4.91 × 10^1^	2.33 × 10^−2^	1.24 × 10^−4^	1.28 × 10^−7^	**3.33 × 10^−9^**
	time	6.08 × 10^−2^	1.07 × 10^−1^	1.47 × 10^−1^	2.28 × 10^−1^	**5.30 × 10^−2^**	9.76 × 10^−2^	1.50 × 10^−1^	1.45 × 10^−1^	1.04 × 10^−1^	1.94 × 10^−1^
	convergence	Yes	Yes	Yes	Yes	Yes	Yes	Yes	Yes	Yes	Yes
	best	1.75 × 10^2^	2.13 × 10^−2^	−2.22 × 10^−16^	2.65 × 10^−13^	1.01 × 10^−8^	−1.11 × 10^−16^	9.14 × 10^−5^	7.69 × 10^−6^	**−2.22 × 10^−16^**	**−2.22 × 10^−16^**
	std.	3.84 × 10^3^	5.06 × 10^0^	7.16 × 10^−6^	5.42 × 10^−6^	1.61 × 10^2^	2.38 × 10^−2^	1.86 × 10^−2^	4.16 × 10^−5^	8.03 × 10^−17^	**6.77 × 10^−17^**
F7	avg.	2.45 × 10^3^	1.28 × 10^0^	2.41 × 10^−6^	2.48 × 10^−6^	5.09 × 10^1^	7.69 × 10^−3^	1.35 × 10^−2^	6.32 × 10^−5^	−1.79 × 10^−16^	**−2.00 × 10^−16^**
	time	1.68 × 10^−1^	2.15 × 10^−1^	3.59 × 10^−1^	4.59 × 10^−1^	**1.56 × 10^−1^**	2.08 × 10^−1^	2.65 × 10^−1^	2.47 × 10^−1^	3.20 × 10^−1^	4.10 × 10^−1^
	convergence	Yes	Yes	Yes	Yes	Yes	Yes	Yes	Yes	Yes	Yes
	best	2.09 × 10^1^	**0.00 × 10^0^**	**0.00 × 10^0^**	**0.00 × 10^0^**	**0.00 × 10^0^**	**0.00 × 10^0^**	**0.00 × 10^0^**	**0.00 × 10^0^**	**0.00 × 10^0^**	**0.00 × 10^0^**
	std.	2.61 × 10^1^	**0.00 × 10^0^**	**0.00 × 10^0^**	**0.00 × 10^0^**	**0.00 × 10^0^**	**0.00 × 10^0^**	**0.00 × 10^0^**	**0.00 × 10^0^**	**0.00 × 10^0^**	**0.00 × 10^0^**
F8	avg.	6.67 × 10^1^	**0.00 × 10^0^**	**0.00 × 10^0^**	**0.00 × 10^0^**	**0.00 × 10^0^**	**0.00 × 10^0^**	**0.00 × 10^0^**	**0.00 × 10^0^**	**0.00 × 10^0^**	**0.00 × 10^0^**
	time	8.55 × 10^−2^	1.36 × 10^−1^	2.10 × 10^−1^	2.65 × 10^−1^	**7.32 × 10^−2^**	1.33 × 10^−1^	1.82 × 10^−1^	1.71 × 10^−1^	1.49 × 10^−1^	2.20 × 10^−1^
	convergence	Yes	No	Yes	Yes	Yes	Yes	No	Yes	No	Yes
	best	6.13 × 10^−10^	8.88 × 10^−15^	9.34 × 10^−218^	**0.00 × 10^0^**	1.07 × 10^−195^	**0.00 × 10^0^**	8.88 × 10^−15^	1.43 × 10^−32^	2.20 × 10^−212^	**0.00 × 10^0^**
	std.	6.02 × 10^−1^	3.82 × 10^−15^	**0.00 × 10^0^**	**0.00 × 10^0^**	4.48 × 10^−15^	2.38 × 10^−51^	**0.00 × 10^0^**	3.07 × 10^−15^	5.27 × 10^−146^	**0.00 × 10^0^**
F9	avg.	1.10 × 10^−1^	1.57 × 10^−14^	3.31 × 10^−187^	**0.00 × 10^0^**	5.03 × 10^−15^	4.34 × 10^−52^	8.88 × 10^−15^	7.70 × 10^−15^	9.63 × 10^−147^	**0.00 × 10^0^**
	time	7.60 × 10^−2^	1.18 × 10^−1^	1.71 × 10^−1^	2.41 × 10^−1^	**6.67 × 10^−2^**	1.06 × 10^−1^	1.59 × 10^−1^	1.47 × 10^−1^	1.13 × 10^−1^	1.90 × 10^−1^
	convergence	Yes	Yes	Yes	Yes	Yes	Yes	Yes	Yes	Yes	Yes
	best	4.91 × 10^1^	9.30 × 10^−3^	6.08 × 10^−210^	5.60 × 10^−8^	7.11 × 10^−15^	9.28 × 10^−5^	7.53 × 10^−4^	6.48 × 10^−4^	1.53 × 10^−198^	**0.00 × 10^0^**
	std.	1.23 × 10^1^	2.32 × 10^1^	4.56 × 10^−4^	4.42 × 10^−5^	1.08 × 10^1^	3.95 × 10^1^	2.48 × 10^1^	2.43 × 10^−4^	2.76 × 10^−111^	**0.00 × 10^0^**
F10	avg.	5.44 × 10^1^	7.02 × 10^1^	1.87 × 10^−4^	6.27 × 10^−5^	4.04 × 10^0^	2.50 × 10^1^	8.05 × 10^0^	1.05 × 10^−3^	5.04 × 10^−112^	**0.00 × 10^0^**
	time	7.72 × 10^−2^	1.15 × 10^−1^	1.76 × 10^−1^	2.34 × 10^−1^	**6.46 × 10^−2^**	1.10 × 10^−1^	1.58 × 10^−1^	1.46 × 10^−1^	1.15 × 10^−1^	1.95 × 10^−1^
	convergence	Yes	Yes	Yes	Yes	Yes	Yes	Yes	Yes	Yes	Yes

**Table 5:** Tension/compression spring design optimization problem.

Algorithms	*d*	*D*	*p*	Best	Std.	Mean
OCBKA	5.0000 × 10^−2^	6.0761 × 10^−1^	2.0000 × 10^0^	**1.2152 × 10^−1^**	4.1658 × 10^−6^	**1.2152 × 10^−1^**
PSO	5.0000 × 10^−2^	6.0761 × 10^−1^	2.0000 × 10^0^	**1.2152 × 10^−1^**	3.0319 × 10^−9^	**1.2152 × 10^−1^**
GWO	5.0000 × 10^−2^	6.0761 × 10^−1^	2.0000 × 10^0^	**1.2152 × 10^−1^**	8.6838 × 10^−5^	1.2160 × 10^−1^
HHO	5.0000 × 10^−2^	6.0761 × 10^−1^	2.0000 × 10^0^	**1.2152 × 10^−1^**	2.9851 × 10^−3^	1.2247 × 10^−1^
GTO	5.0000 × 10^−2^	6.0761 × 10^−1^	2.0000 × 10^0^	**1.2152 × 10^−1^**	2.9257 × 10^−17^	**1.2152 × 10^−1^**
SO	5.0000 × 10^−2^	6.0761 × 10^−1^	2.0000 × 10^0^	**1.2152 × 10^−1^**	2.1474 × 10^−2^	1.3410 × 10^−1^
DBO	5.0000 × 10^−2^	6.0761 × 10^−1^	2.0000 × 10^0^	**1.2152 × 10^−1^**	**2.8138 × 10^−17^**	**1.2152 × 10^−1^**
GJO	5.0000 × 10^−2^	6.0759 × 10^−1^	2.0000 × 10^0^	1.2153 × 10^−1^	1.7488 × 10^−4^	1.2167 × 10^−1^
SABO	5.0000 × 10^−2^	6.1232 × 10^−1^	2.0198 × 10^0^	1.2307 × 10^−1^	1.9114 × 10^−2^	1.3895 × 10^−1^
BKA	5.0000 × 10^−2^	6.0761 × 10^−1^	2.0000 × 10^0^	**1.2152 × 10^−1^**	1.2616 × 10^−6^	**1.2152 × 10^−1^**

**Table 6:** Results of solving the optimization problem for the three-bar truss design.

Algorithms	*X_1_*	*X_2_*	Best	Std.	Mean
OCBKA	7.6494 × 10^−1^	3.9596 × 10^−1^	**2.5981 × 10^2^**	7.0524 × 10^−10^	**2.5981 × 10^2^**
PSO	7.6494 × 10^−1^	3.9596 × 10^−1^	**2.5981 × 10^2^**	1.1449 × 10^−6^	**2.5981 × 10^2^**
GWO	7.6523 × 10^−1^	3.9524 × 10^−1^	**2.5981 × 10^2^**	2.6145 × 10^−3^	**2.5981 × 10^2^**
HHO	7.6466 × 10^−1^	3.9675 × 10^−1^	**2.5981 × 10^2^**	4.8737 × 10^−1^	2.6012 × 10^2^
GTO	7.6494 × 10^−1^	3.9596 × 10^−1^	**2.5981 × 10^2^**	**1.2358 × 10^−12^**	**2.5981 × 10^2^**
SO	7.6492 × 10^−1^	3.9600 × 10^−1^	**2.5981 × 10^2^**	4.9399 × 10^−4^	**2.5981 × 10^2^**
DBO	7.6494 × 10^−1^	3.9597 × 10^−1^	**2.5981 × 10^2^**	1.7585 × 10^−5^	**2.5981 × 10^2^**
GJO	7.6485 × 10^−1^	3.9658 × 10^−1^	**2.5981 × 10^2^**	5.2992 × 10^−3^	**2.5981 × 10^2^**
SABO	7.6670 × 10^−1^	3.9685 × 10^−1^	2.5983 × 10^2^	2.5174 × 10^−1^	2.6003 × 10^2^
BKA	7.6494 × 10^−1^	3.9596 × 10^−1^	**2.5981 × 10^2^**	7.6086 × 10^−8^	**2.5981 × 10^2^**

**Table 7:** Comparison results for the speed reducer optimization design problem.

Algorithms	*X_1_*	*X_2_*	*X_3_*	*X_4_*	*X_5_*	*X_6_*	*X_7_*	Best	Std.	Mean
OCBKA	2.7666 × 10^0^	7.0000 × 10^−1^	1.7000 × 10^1^	7.3000 × 10^0^	7.3000 × 10^0^	3.2288 × 10^0^	5.0000 × 10^0^	**2.6388 × 10^3^**	1.5086 × 10^−2^	**2.6388 × 10^3^**
PSO	2.7666 × 10^0^	7.0000 × 10^−1^	1.7000 × 10^1^	7.3000 × 10^0^	7.3000 × 10^0^	3.2288 × 10^0^	5.0000 × 10^0^	**2.6388 × 10^3^**	6.8153 × 10^0^	2.6418 × 10^3^
GWO	2.7543 × 10^0^	7.0000 × 10^−1^	1.7000 × 10^1^	7.3000 × 10^0^	7.3000 × 10^0^	3.2314 × 10^0^	5.0000 × 10^0^	2.6389 × 10^3^	3.6336 × 10^0^	2.6421 × 10^3^
HHO	2.7676 × 10^0^	7.0000 × 10^−1^	1.7000 × 10^1^	7.3000 × 10^0^	7.3000 × 10^0^	3.2283 × 10^0^	5.0000 × 10^0^	**2.6388 × 10^3^**	6.3292 × 10^0^	2.6417 × 10^3^
GTO	2.7666 × 10^0^	7.0000 × 10^−1^	1.7000 × 10^1^	7.3000 × 10^0^	7.3000 × 10^0^	3.2288 × 10^0^	5.0000 × 10^0^	**2.6388 × 10^3^**	8.7254 × 10^0^	2.6430 × 10^3^
SO	2.7666 × 10^0^	7.0000 × 10^−1^	1.7000 × 10^1^	7.3000 × 10^0^	7.3000 × 10^0^	3.2288 × 10^0^	5.0000 × 10^0^	**2.6388 × 10^3^**	6.8258 × 10^0^	2.6418 × 10^3^
DBO	2.7666 × 10^0^	7.0000 × 10^−1^	1.7000 × 10^1^	7.3000 × 10^0^	7.3000 × 10^0^	3.2288 × 10^0^	5.0000 × 10^0^	**2.6388 × 10^3^**	6.4565 × 10^0^	2.6416 × 10^3^
GJO	2.8136 × 10^0^	7.0000 × 10^−1^	1.7000 × 10^1^	7.3000 × 10^0^	7.3000 × 10^0^	3.2570 × 10^0^	5.0000 × 10^0^	2.6412 × 10^3^	6.7248 × 10^0^	2.6491 × 10^3^
SABO	2.7080 × 10^0^	7.0000 × 10^−1^	1.7000 × 10^1^	7.3000 × 10^0^	7.6703 × 10^0^	3.1565 × 10^0^	5.0000 × 10^0^	2.6556 × 10^3^	1.1918 × 10^1^	2.6674 × 10^3^
BKA	2.7667 × 10^0^	7.0000 × 10^−1^	1.7000 × 10^1^	7.3000 × 10^0^	7.3000 × 10^0^	3.2287 × 10^0^	5.0000 × 10^0^	**2.6388 × 10^3^**	**7.7086 × 10^−3^**	**2.6388 × 10^3^**

## Data Availability

The data that support the findings of this study are available from the corresponding author upon request. There are no restrictions on data availability.
